# Development of the Hearts of Lizards and Snakes and Perspectives to Cardiac Evolution

**DOI:** 10.1371/journal.pone.0063651

**Published:** 2013-06-05

**Authors:** Bjarke Jensen, Gert van den Berg, Rick van den Doel, Roelof-Jan Oostra, Tobias Wang, Antoon F. M. Moorman

**Affiliations:** 1 Department of Bioscience – Zoophysiology, Aarhus University, Aarhus, Denmark; 2 Department of Anatomy, Embryology & Physiology, Academic Medical Center, University of Amsterdam, Amsterdam, The Netherlands; New York Medical College, United States of America

## Abstract

Birds and mammals both developed high performance hearts from a heart that must have been reptile-like and the hearts of extant reptiles have an unmatched variability in design. Yet, studies on cardiac development in reptiles are largely old and further studies are much needed as reptiles are starting to become used in molecular studies. We studied the growth of cardiac compartments and changes in morphology principally in the model organism corn snake (*Pantherophis guttatus*), but also in the genotyped anole (*Anolis carolinenis* and *A. sagrei*) and the Philippine sailfin lizard (*Hydrosaurus pustulatus*). Structures and chambers of the formed heart were traced back in development and annotated in interactive 3D pdfs. In the corn snake, we found that the ventricle and atria grow exponentially, whereas the myocardial volumes of the atrioventricular canal and the muscular outflow tract are stable. Ventricular development occurs, as in other amniotes, by an early growth at the outer curvature and later, and in parallel, by incorporation of the muscular outflow tract. With the exception of the late completion of the atrial septum, the adult design of the squamate heart is essentially reached halfway through development. This design strongly resembles the developing hearts of human, mouse and chicken around the time of initial ventricular septation. Subsequent to this stage, and in contrast to the squamates, hearts of endothermic vertebrates completely septate their ventricles, develop an insulating atrioventricular plane, shift and expand their atrioventricular canal toward the right and incorporate the systemic and pulmonary venous myocardium into the atria.

## Introduction

**Figure 1:**
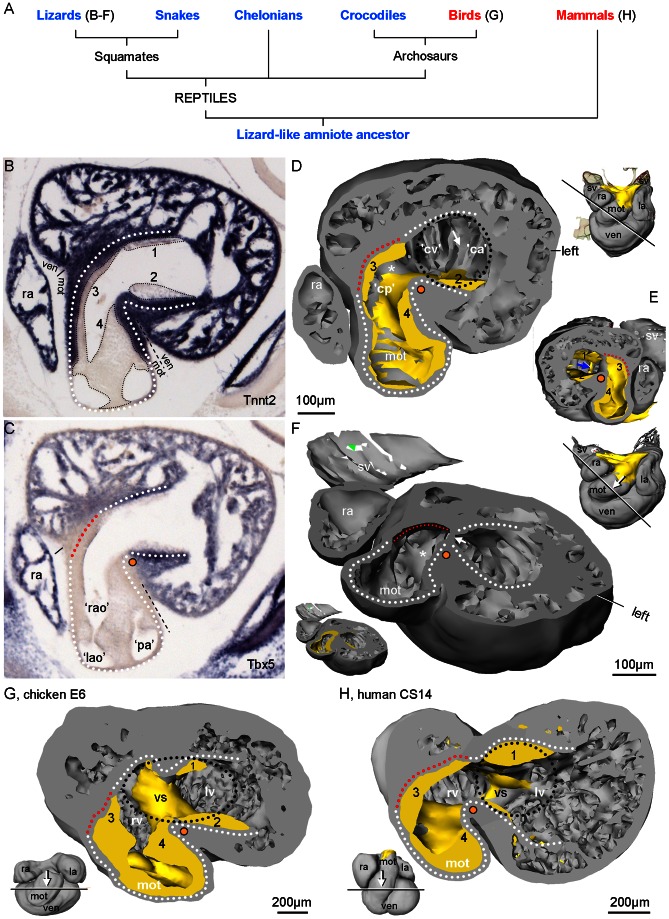
Terminology of the ventricle based on *Anolis sagrei* St9/19 embryos. **A**. Phylogenetic tree of amniote evolution, with ectotherms in blue and endotherms in red. Reptilia includes birds but we use ‘reptile’to mean ectothermic members of Reptilia. **B**. A 10 µm thick section, close to the transverse plane, stained for *Anolis* cardiac troponin 2. Smooth-walled myocardium (white stipulated lines) extends from the otherwise much trabeculated ventricle (ven) to the myocardial outflow tract (mot) and is reminiscent of the earlier heart tube myocardium. Mesenchyme is associated with the smooth-walled myocardium and can be categorized as dorsal (1) and ventral (2) atrioventricular cushion and parietal (3) and septal (4) cushion (nomenclature of the mammalian heart [53]. **C**. Sister section to A stained for *Anolis Tbx5*, which is absent from the mot in all vertebrates. The mot is incorporated to the ventricle during development, which creates the bulbuslamelle (red stipulated line) and a fold in the ventricular wall also known as the muscular ridge (orange dot). **D**. A 3D reconstruction, of a different specimen, cut in a plane corresponding to the sections of B–C (mesenchyme in yellow). It shows the complete part of the muscular ridge (asterisk) and its free-standing part (orange dot). Oppositely, the bulbuslamelle is found and notice the associated cushion. The position of the atrioventricular canal is indicated by the black stipulated line, the probable position of the future vertical septum is indicated with the white arrow and the positions of the future cavum arteriosum (‘ca’), cavum venosum (‘cv’) and cavum pulmonale (‘cp’) can accordingly be designated. **E**. Same sectioning plane as in C, looking towards the atria, with the atrial septum indicated (blue arrow) **F**. Slightly more tilted sectioning plane than in C looking towards the deeper right parts of the muscular ridge (asterisk) from a skewed angle (the mesenchyme is removed, but shown in the miniature). Also shown, is where the atrioventricular canal meets the ventricle (arrow). **G**. Chicken hearts of Hamburger/Hamilton stage 28–29 (embryonic day 6) also have a reptilian-like design (modified from [23]). **H**. Human heart, Carnegie stage 14 (embryonic days 31–35) has essentially the same design as the reptilian heart, with the ventricular septum (vs) being an important variation. Notice that the bulbuslamelle (red stipulated line) is defined by the presence of mesenchyme (modified from [51]). l(r)a, left(right) atrium; sv, sinus venosus.

Mammals and the common lineage of birds and crocodilians (the archosaurs) are the only vertebrates where the cardiac ventricle has complete anatomical division of the systemic and pulmonary sides. Both mammals and birds stem from a lizard-like ancestor (Fig. 1) and while all seem to agree that the divided hearts of mammals and birds evolved independently from undivided reptile-like hearts [1], [2] several competing hypotheses seek to identify the primordial structures of the reptile ventricle that gave rise to the advanced state of mammals and birds. Some studies emphasize a right-sided partial septum called the muscular ridge [1], [3], while others emphasize a left-sided trabeculation (the vertical septum) because of its proximity to the atrioventricular valve [4]. The validity of these competing hypotheses remains difficult to assess because little is known about the embryological development of the reptilian heart.

The fully formed heart of non-crocodilian reptiles (*i.e.* squamates and chelonians) receives inflow from three systemic veins to the right atrium as well as one or two pulmonary veins that feeds into a single orifice to the left atrium, while the outflow occurs through three arteries, the left and right aortae and a single pulmonary artery [5]. The two atrial chambers are fully separated, and the sinus venosus is situated upstream of the right atrium. In diastole the ventricular receives blood from the left atrium to its left-most compartment, the cavum arteriosum, whereas blood of the right atrium is received in central cavum venosum and the right-most compartment, the cavum pulmonale. The right atrial blood is then predominantly directed towards the pulmonary artery and the left atrial blood towards the aortae. In most reptiles, however, the ventricle is not divided into a low pressure right ventricle and a high pressure left ventricle, but functions as a single pressure pump [6]. It is therefore pulmonary to systemic outflow resistances that determine where the ventricular blood is ejected to. Resistance is typically highest in the pulmonary circulation, at least in resting animals, and cardiac output is thus disproportionally directed to the systemic circulation, a so-called right-to-left shunt. Blood flows are nonetheless well separated within the ventricle, probably due to the septa [5]–[9], and admixture of oxygen-poor and oxygen-rich blood is minimized. Being ectothermic, the cardiac output, heart rate and blood pressures of reptiles are generally much lower than in the endothermic mammals and birds, but similar to those of amphibians [9]–[11].

The ventricle is anatomically the most complex chamber and the nomenclature of the structures and compartments are introduced in Figure 1. Much of our results will be discussed in the context of cardiac evolution, so we extend the nomenclature to avian and mammalian hearts (Fig. 1G–H). Also, we provide a glossary of the described structures, which includes definitions and synonyms. There is no standardized nomenclature on embryonic cardiac structures for amniotes. Because we focus on reptiles, we adopt the nomenclature of previous works on reptile cardiac development, which, unfortunately, is not standardized as well. Figure 1 is based on anole specimens halfway through development where many early structures are still distinct and the fully formed heart is outlined. The formed squamate heart is partially divided into three compartments, called cava, by three structures, usually referred to as septa. From left to right, the cava are the cavum arteriosum, the cavum venosum and the cavum pulmonale. The cavum arteriosum is partially separated from the cavum venosum by a sheet-like aggregation of trabeculations called the vertical septum. The cavum venosum is partially separated from the cavum pulmonale by a spiraling septum called the muscular ridge (also known as the horizontal septum [3], [12]), Muskelleiste [13] or other names [5]). Exactly opposite the muscular ridge is the bulbuslamelle *e.g.* [14] (‘Bulbuslamelle’was coined by Greil [15] instead of Brückes name of ‘Fleischpolster’ [16]). Very little is known about when and how these structures appear in the ontogenetic development of any reptile.

The few embryological studies on reptile hearts are in stark contrast to the vast number of studies of cardiac development in fish, amphibians, birds and mammals [4], [11], [17], [18]. Nonetheless, the embryology of reptile hearts remains pivotal for our understanding of vertebrate cardiac evolution [4], [12], [15], [19]–[23]. For instance, Greil [15] argued that the myocardial outflow tract contributes to the right ventricle in reptiles, mammals and birds and this has been verified experimentally multiple times by gene expression, cell fate and lineage tracings [24]–[26]. Here, we describe the growth and morphological changes after the early heart tube formation to the formed heart in the corn snake (*Pantherophis guttatus guttatus*) and in the green and brown anoles (*Anolis carolinensis* and *A. sagrei*). Genome sequencing was recently completed in the green anole [27] and is underway in the corn snake [28], which can be expected to greatly facilitate gene expression studies.

This study provides a developmental series from the onset of chamber formation of some of the most commonly used squamate models, the corn snake and the anole. The reptile heart and the ventricle in particular have a very complex anatomy and we have therefore provided comprehensively annotated 3D models in pdf format of all investigated stages of the corn snake from 2 to 42 days after egg laying. Secondly, we discuss separately the findings in the context of cardiac evolution. It is the ventricular anatomy that varies the most between reptiles, mammals and birds. This study emphasizes the ventricle.

## Materials and Methods

### Specimens

Fertilized eggs of the corn snake (*P. guttatus guttatus*) and two fertilized eggs of the Philippine sailfin lizard (*Hydrosaurus pustulatus*) were incubated on gravel to prevent contact with water in an incubator at 29°C and 85% humidity. Fertilized eggs of the green and brown anoles (*Anolis carolinensis* and *A. sagrei*) were bought commercially and fixed immediately in a 4% paraformaldehyde phosphate-buffered saline solution overnight and then 70% ethanol followed by embedding in paraplast. Corn snake embryos were fixed similarly at 2, 10, 12, 14, 16, 20, 26, 35 and 42 days post egg laying (referred to as days) out of ca. 60 days, sailfin lizard embryos at 4 and 7 days out of ca. 60 days and anoles at stages 5, 7, 9, 12 and 17 out of 19 (anole development, from egg laying, lasts ca. 25 days and includes stages 4–19, [29]). The hearts of a 3 months-old corn snake and an adult green anole, ca. 1 year, were included as fully formed hearts. A heart of an adult Burmese python and an adult ostrich were included in the comparative analyses. All adult hearts were fixed as above.

### Ethics statement

In The Netherlands experiments with non-mammalian embryos (that are not autonomously viable) do not require approval from the Institutional Animal Care and Use Committee. The fertilized corn snake and sailfin lizard eggs were donated to us (R-JO) from the Diergaarde Blijdorp (Rotterdam, the Netherlands) whereas fertilized anole lizard eggs were obtained commercially. All embryos were sacrificed by immersion in 4% paraformaldehyde except in the late developmental stages, corn snake 42 days and anole lizard stages 17 and 19, where the embryo was first decapitated and the head split saggittally to stop all brain activity. Adult reptiles were commercially obtained and sacrificed in Denmark in accordance with Danish Federal Regulation, where the harvest of tissue of sacrificed animals by anesthesia does not require approval from Institutional Animal Care and Use Committee; the animals were anaesthetized with 100 mg pentobarbital (Sygehusapotekerne, Denmark) per kilo body mass and then had their hearts excised. The carcass of a euthanized ostrich was given to us (BJ and TW) by Givskud Zoo, Denmark.

### Sectioning, staining, MRI scanning and 3D reconstructions

Most specimens were cut in 10 µm sections, except corn snakes of 26, 35 and 42 days and 3 months that were cut in 14 µm sections and the adult green anole which was cut in 12 µm sections. Myocardial staining was performed as previously described [30]. In all specimens of corn snake and sailfin lizard the myocardium was visualized by immunohistochemistry using a rabbit antibody to cardiac troponin I (cTnI) polyclonal antibody (HyTest ltd., dilution 1:500) binding of which was visualized by a fluorescently labelled secondary goat-anti-rabbit antibody coupled to Alexa 568 (Invitrogen, dilution 1:250). Two specimens of anole (stage 5 and 12) were cut in 7 µm sections and stained for the myocardial marker, cTnI, as described above, and were additionally stained for all nuclei with Sytox Green (1:40,000 Molecular Probes S-7020), and for incorporation of Bromodeoxyuridine (BrdU), a synthetic analogue of thymidine used to detect DNA replication, with a rat-monoclonal anti-BrdU (1:600, Immunosource). Antibody binding was then visualized using a fluorescently labelled secondary goat-anti-rat antibody coupled to Alexa 680 (Invitrogen, dilution 1:250). 100 µl BrdU (10 mg BrdU (Sigma) per ml physiological salt solution (0.9% NaCl)) was injected through the shell into the egg yolk. Incubation with BrdU was at room temperature for one hour.

**Figure 2:**
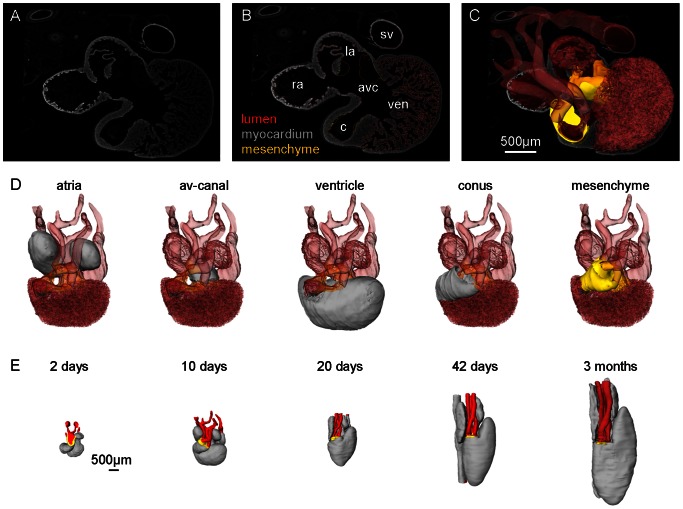
Generation of 3D reconstructions. A. Example of a 10 µm section from *P. guttatus* 10 days stained for myocardium with antibodies against rabbit cardiac troponin I. B. Same section as in A with annotations made in Amira®. C. 3D reconstruction based on 160 sections projected out of the section of A with mesenchyme and transparent lumen (myocardium not shown). D. Same 3D reconstruction as in C visualizing the annotated compartments. E. Five reconstructions exemplifying the transition from the youngest to the oldest stage.

We used *in-situ* hybridizations for *Tnnt2* (anole cardiac troponin T) for myocardial stain and *Tbx5* on anole stages 7, 9 and 17 [23]. Only the adult green anole heart was stained with picro-sirius red for collagen. All sections were photographed, stacked, and aligned in Amira® v4.1.1 (or newer versions) and then reconstructed as previously described (Fig. 2A–C) [31], [32]. In Amira® we annotated the developing compartments on the following morphological criteria; the myocardial outflow tract is the smooth-walled tract of the arterial pole; the ventricle is the trabeculated chamber upstream of the myocardial outflow tract and downstream of the atrioventricular canal; the atrioventricular canal is the smooth-walled cylindrical canal in between the more voluminous atria and ventricle; the atria are the compartment between the atrioventricular canal and the sinus venosus, i.e. the systemic inflows to the right atrium with myocardium (Fig. 2D). By using the MaterialStatistics tool in Amira® we obtained volume readouts for all annotated structures. Length of the myocardial outflow tract was measured as described previously [33].

In our discussion of the evolutionary fates of the bulbuslamelle we compare to python and bird hearts on the basis of 3D models made from MRI scans. The MRI scans were performed with clinically available Philips Achieva 1.5 T system (Philips Medical Systems, Amsterdam, the Netherlands). The hearts were imbedded in agar and positioned in the centre of the magnet. Data were acquired with a dedicated radiofrequency surface coil using high-resolution 3D gradient–echo sequence with the following parameters: *Python molurus*; field-of-view: 60×80×80 mm^3^; repetition time: 75 ms; echo time: 5.4 ms and excitation flip angle: 45°. Images were isotropically acquired with a spatial resolution of 0.9×0.9×0.9 mm^3^/voxel. Ostrich; field-of-view: 230×230×140 mm^3^, voxel size: 0.48×0.48×0.48 mm^3^, repetition time: 15.1 ms, echo time: 6.9 ms, excitation flip angle: 30°. Reconstructed images were exported in DICOM format and loaded to Amira and treated as above.

Amira models were converted to interactive 3D pdfs using Adobe Acrobat Pro Extended® version 9.3 as previously described [34]. The 3D pdf can be viewed with the freeware version: Adobe Reader® (version 9.3 or higher) with Javascript® enabled.

## Results

### 3D reconstructions in interactive pdfs

The cardiac lumen and the following structures of the corn snake heart are annotated in the supplementary 3D pdfs based on morphology; sinus venosus, sinuatrial valves (10 days onwards), septum spurium, atria (including atrial septum), atrioventricular canal, ventricle, bulboauricularlamella (10 days onwards), bulbuslamelle (10 days onwards), bulbo-ventricular fold (2 days)/muscular ridge (10 days onwards), vertical septum (20 days onwards) and myocardial outflow tract. The Amira® files, which the 3D pdfs (Figs. S1–7) are based on, are available on request and include the editable label file and the surface file.

### Supplementary videos

We recorded the beating hearts of the corn snake from days 10 and 20 with emphasis on the systemic inflow to the heart and the sequence of chamber contractions and these videos are included in the online material (Movies S1-S6).

### Total cardiac growth

**Figure 3:**
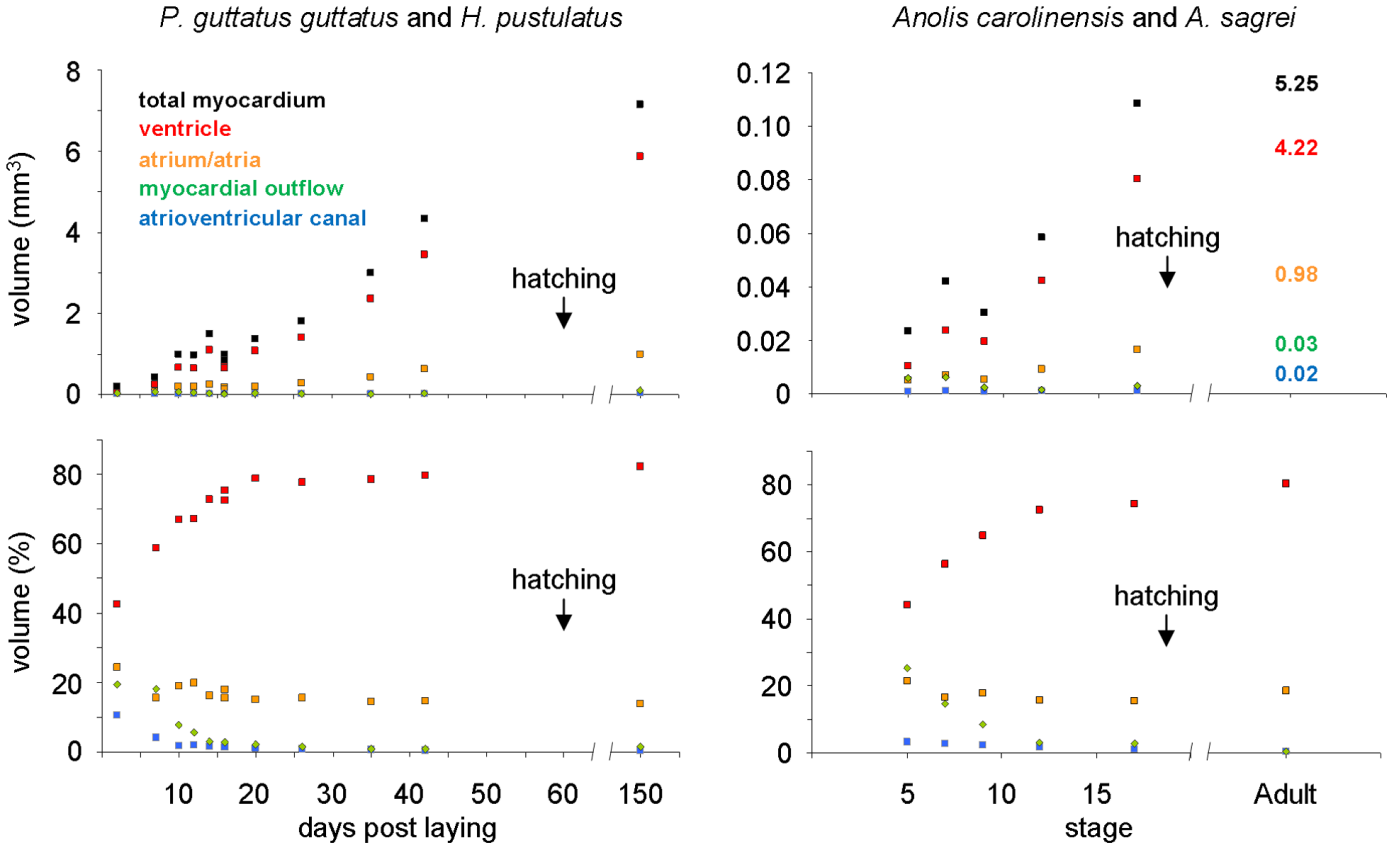
Absolute (mm^3^) and relative (%) myocardial volumes of cardiac compartments rendered from 3D reconstructions. Myocardial volume of the ventricle and the atria increases exponentially. Neither the conus nor the atrioventricular canal changes substantially in size and come to contribute only miniscule proportions to the fully formed hearts represented by 150 days for the corn snake (3 months post hatching) and ‘Adult’ for the anoles (the mm^3^ values for the adult anole are written onto the graph).

In the earliest stage available, *i.e.* 2 days in the corn snake, chamber formation, *i.e.* the ballooning of ventricular and atrial compartments, was already initiated (Fig. 2E). At this point the myocardial volume is 0.2 µl, which by 42 days has increased exponentially and more than 20 fold to a volume of 4.3 µl (Fig. S8), whereas some 100 days later, 3 months after hatching (*i.e.* juvenile snake), this volume has less than doubled to 7.2 µl (Fig. 3). In the anoles, the heart of stage 5/19 is morphologically similar to the corn snake 2 days, but it is much smaller, 0.024 µl. It grows ca. 5 fold before hatching (st17/19), to 0.108 µl, and then almost 50 fold to reach the adult condition (5.25 µl) (Fig. 3). Cardiac growth is accompanied by an expansion of the pericardial cavity. The distance from the pericardial wall to the atria and the conus arteriosus respectively increases and vessels form accordingly.

### Growth of compartments

All compartments found in the adult heart can be recognized by 2 days in the corn snake and st5/19 in the anoles. We annotated and measured the volumes of the myocardial outflow tract, the ventricle, the atrioventricular canal, the atrium (later atria), the sinus venosus and mesenchymal tissue throughout development in both the corn snake and the anoles. Only the sinus venosus myocardium was often damaged and thus incompletely reconstructed. As described for mammals and birds, there are clear compartmental differences in growth [35], [36]. The myocardial volumes of the atrioventricular canal and myocardial outflow tract were essentially constant throughout development, whereas both ventricular and atrial compartments increased exponentially (Fig. 3, Fig. S8). In the two anole specimens exposed to BrdU, an indicator of proliferation, BrdU was incorporated at the highest rates in the regions of exponential growth, *i.e.* the atria and ventricle (Fig. S9). In the corn snake, the atrioventricular and ventricular mesenchyme, which in later stages remodel into the atrioventricular (ca. 35 days) and arterial valves (ca. 35 days), constituted 0.1–0.15 µl throughout development and 0.17 µl at three months after hatching. By 20 days in the corn snake, one-third through development, the ventricle and atria constituted ca. 80% and ca. 15% respectively of the total myocardial volume which is essentially the proportions of the 3 months heart (Fig. 3). Halfway through development in anoles, st12/19, the proportions of the ventricle and atria (72,6% and 15,8% respectively) components approximates that of the adult (80,5% and 18,6% respectively, Fig. 3).

**Figure 4:**
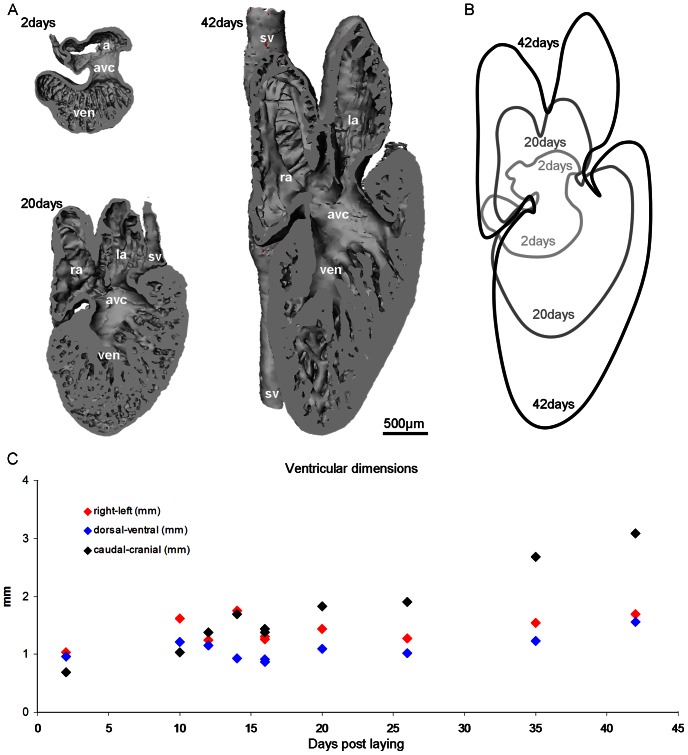
Changes in cardiac dimensions throughout development in the corn snake. A. To scale reconstructions, showing the dorsal halve of the heart.. B. Cardiac profiles during development, arbitrarily fixed at the right margin of the atrioventricular canal. C. The ventricle is initially wider (right-left) and deeper (dorsal-ventral) than long (caudal-cranial), but growth is primarily by lengthening. avc, atrioventricular canal; l(r)a, left(right) atrium; sv, sinus venosus; ven, ventricle.

The hearts mature towards the adult shape throughout development. This is particularly evident in corn snakes, where the heart becomes more elongate than the anole heart (Fig. 4). At 2 days the corn snake heart is 1.2 mm long (caudo-cranially) and 1 mm wide (from left to right). By 20 days, where the atrial and ventricular compartments have the proportional volume of the adult (Fig. 3), the heart is still stockier than the heart at 42 days, which is more than twice as long as wide, 4,5 mm to 1,9 mm, respectively (Fig. 4). From 2 days to 42 days the corn snake ventricle expands less than 2 fold from left to right and dorso-ventrally, but expands more than 4 fold caudo-cranially (Fig. 4).

### Appearance of structures

The appearance of structures is listed chronologically for the corn snake, the sailfin lizard and the anoles in Table 1. We deem that development in the corn snake of 2 to 14 days and in the anoles of stages 5 to 9 correspond to 3 to 6 days of chicken development (Hamburger-Hamilton stages 18–28 [37]) and Carnegie stages 14 to 18 of human development. By Hamburger-Hamilton stage 28, the developing chicken heart has an external resemblance to that of corn snake day 14 and anole st9/19 but internally the ventricular septum, which does not develop in squamates, is already well-defined. In the following sections the development of each compartment will be treated.

**Table 1:** Chronological appearance of cardiac structures.

Days	Corn snake	Sailfin lizard
2	sinus venosus	
	single atrium	
	ventricular trabeculation	
	bulbo-ventricular fold	
4		ventricular trabeculation
		bulbo-ventricular fold
		endocardial atrial septum
7		sinuatrial valves
10	pulmonary vein	
	myocardial atrial septum	
	septum spurium	
	bulboauricularlamella	
14	settled position of SA-orifice	
	cavum pulmonale	
	muscular ridge	
	bulbuslamelle	
20	vertical septum	
	cavum arteriosum	
	cavum venosum	
∼60	hatching	
**Stage**	**Anoles**	
5	sinus venosus	
	ventricular trabeculation	
	bulbo-ventricular fold	
	endocardial atrial septum	
	sinuatrial valves	
7	pulmonary vein	
	myocardial atrial septum	
	septum spurium	
9	bulboauricularlamella	
	cavum pulmonale	
	muscular ridge	
	bulbuslamelle	
12	vertical septum	
	cavum arteriosum	
	cavum venosum	
		
19	hatching	

The corn snake and the sailfin lizard have been aligned because both have similar incubation times after egg laying. In comparing squamate heart development to other vertebrates, the developing hearts of the corn snake of 2 to 14 days and the anoles of stages 5 to 9 correspond in many regards to, we suggest, the hearts of chicken development of Hamburger-Hamilton stages 18–28 and of human development of Carnegie stages 14 to 18. Later stages differ substantially.

### Inflow to the heart

There is initially a very short distance from the entry of the systemic veins through the pericardial wall to the (right) atrium, but it increases many fold during development. Accordingly, vessels form and elongate in between and they are referred to as the sinus venosus when they acquire cardiac musculature. The sinus venosus initially (2 days) consists of the left and right (anterior) sinus horns. Later, ca. 12–16 days, a third vessel appears which stems from the liver and is confluent with the sinus horns immediately upstream of the right atrium. Video recordings show sequential contractions in the sinus venosus, the atria and the ventricle (Movies S1-S6) and sinuatrial valves are present at this point. The sinuatrial valves are myocardial, contrary to the mesenchymal valves of the atrioventricular canal and arteries. The cardiac musculature encloses the vessels throughout development and only in the left sinus horn does the myocardial enclosure become discontinuous by 42 days.

The future pulmonary vein is indicated by 7 days by a mesenchymal cushion associated with the dorsal wall of the atrium and from 10 days onward a lumen can be seen within the cushion. Unlike mammals, no myocardium develops around the pulmonary vein. By 7 days, sinuatrial valves have started to form, protruding into the atrial lumen.

### The atria

Initially, the atrium is a smooth-walled chamber encompassing a single lumen. The lateral walls of the atrium seemingly outgrow the roof and a deep sulcus is eventually created between the left and right atrium (Fig. 4). Internally, trabeculations (commonly called pectinate muscles or *musculi pectinati*) appear by 10 days, the most prominent of which is the septum spurium of the right atrium which is continuous with the sinuatrial valves. The atrial septum has also started to form at 10 days but is not completed by 42 days. The atrial septum develops slightly to the left of the body midline such that a small cul-de-sac is formed to the right atrium to the left of the deepest part of the sulcus of the atrial roof. Eventually, by 42 days, the atrial walls are entirely trabeculated except immediately above the atrioventricular canal, *i.e.* the atrial floor, or vestibule.

The atrioventricular canal initially is a free standing tube, but becomes progressively enclosed by ventricular myocardium (Fig. 1A–B, 2E). Already at 2 days of development, the entire atrioventricular canal is lined with mesenchyme, which is particularly thick dorsally and ventrally and thus forming two cushions with a single narrow lumen centrally. Laterally the mesenchyme has disappeared by 10 days and only the two cushions remain. By 16 days the two cushions have fused whereby separate left and right inflows to the ventricle are created. A lateral cushion is seen transiently on the right side of the atrioventricular canal around 20 days but not much remains by 3 months. At no point is the atrioventricular canal myocardium interrupted by fibro-fatty tissue from the atrioventricular sulcus (as seen in mammalian and avian development).

### The ventricle

**Figure 5:**
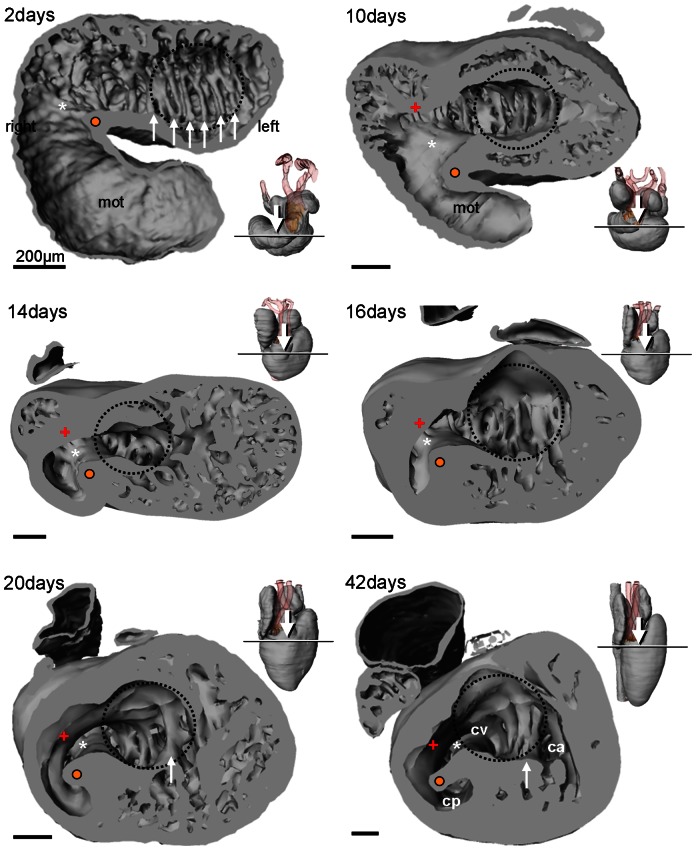
Ventricular morphology of the corn snake. Looping of the heart tube leaves a fold (white asterisk and orange dot), the bulbo-ventricular fold, on the border of the ventricle and the myocardial outflow tract (mot) (2–10 days). Later, and associated with the ventricularization of the mot, this fold constitutes the muscular ridge (14 days and later), with a free standing part (orange dot) and a complete part (white asterisk). Opposite the muscular ridge, the bulbuslamelle forms (+). The early ventricle has multiple parallel trabecular sheets (white arrows) of equal height but as the ventricle grows (at 20 and 42 days) only one sheet retains a short distance to the atrioventricular canal (indicated by the broken blue line) which may then be annotated as the vertical septum. Inserts show sectioning plane and angle of inspection.

The ventricle develops in between a left-sided atrioventricular canal and a right-sided conus orifice. Trabeculations develop in the entire ventricle, except towards the atria and in between the atrioventricular canal and the conus orifice and this smooth-walled part is referred to as the inner curvature. The early trabeculations (2 days) are congregated into multiple trabecular sheets that are parallel to the saggital plane (Fig. 5). Already by 10 days, however, most of the ventricular trabeculations appear as a spongy meshwork in between the sheets that remain centrally and the outer ventricular surface. The sheets are retained throughout development but only one or a few remain at a short distance to the atrioventricular canal from ca. 20 days onwards. If a single sheet is identified this can constitute the vertical septum with the cavum arteriosum to its left and the cavum venosum on its right (Fig. 5).

Formation of the cavum pulmonale probably occurs as a consequence of the ventricularization of the conus and by 14 days a shallow cavum pulmonale appears that will become deeper as the ventricle continues to elongate. The early ventricle therefore appears to consist of the cavum arteriosum and cavum venosum and later, from ca. 14 days onward, the cavum pulmonale develops (Fig. 5). Concomitantly with the deepening of the cavum pumonale, the muscular ridge also elongates in caudo-cranial direction. In earlier stages, the muscular ridge appears as a (bulbo-ventricular) fold in between the early ventricle and the myocardial outflow tract (Fig. 5).

**Figure 6:**
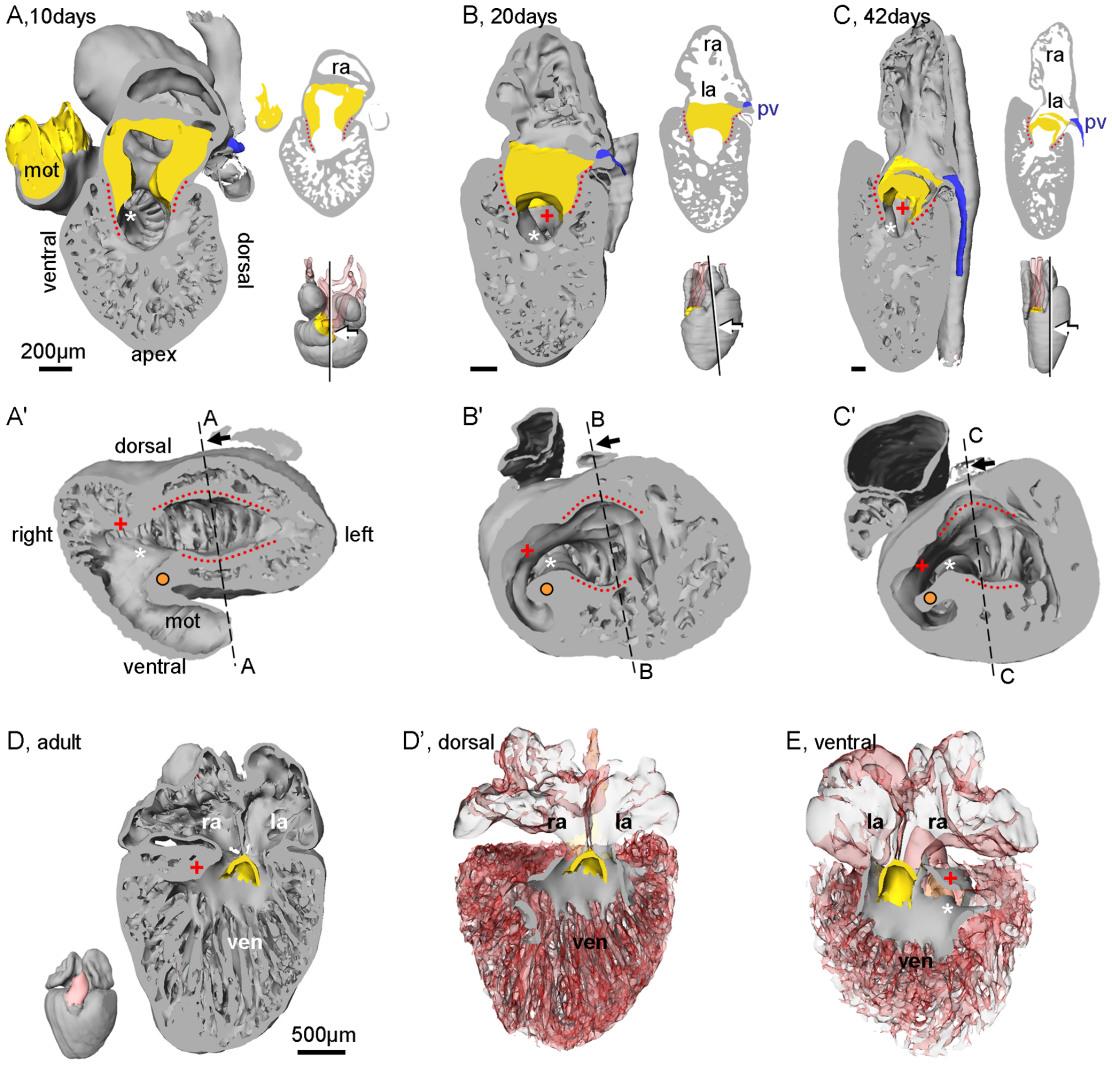
Bulboauricularlamella. Smooth-walled ventricular myocardium, the bulboauricularlamella, is formed early in development (**A**–**C**, corn snake) and remains in the adult heart (**D**–**E**, anole). **A**. The bulboauricularlamella, ventral and dorsal (red dotted lines), are contiguous with the atrioventricular canal (contains mesenchyme, shown in yellow). Inserts show plane of sectioning, angle of inspection and the sectioned plane. **B**–**C**. Once formed, the bulboauricularlamella remains a constant feature of the ventricle. **D**. Dorsal halve of the heart of an adult green anole (atrioventricular valve in yellow). **D'**. Same view as in D with all structures made transparent except the smooth-surfaced myocardium of the ventricle, the majority of which is the dorsal bulboauricularlamelle. **E**. Ventral halve of the same heart, slightly rotated, showing the ventral bulboauricularlamelle and how it blends, without a boundary, into the muscular ridge (askerisk). +, bulbuslamelle; l(r)a, left(right) atrium; orange dot; bulbo-ventricular fold/free-standing part of the muscular ridge; pv, pulmonary vein; ven, ventricle.

From about 10 days onward smooth-walled myocardium associated with the atrioventricular cushions appears to project into the ventricle from the atrioventricular canal ventrally and dorsally and these are called the bulboauricularlamella (Fig. 6) [15]. In the 3D pdfs they are shown to extend to the inner curvature. To the right of the inner curvature smooth-walled myocardium is then regarded as the bulbuslamelle if it is continuous with the dorsal bulboauricularlamelle and the muscular ridge if it is continuous with the ventral bulboauricularlamelle.

### The myocardial outflow tract

**Figure 7:**
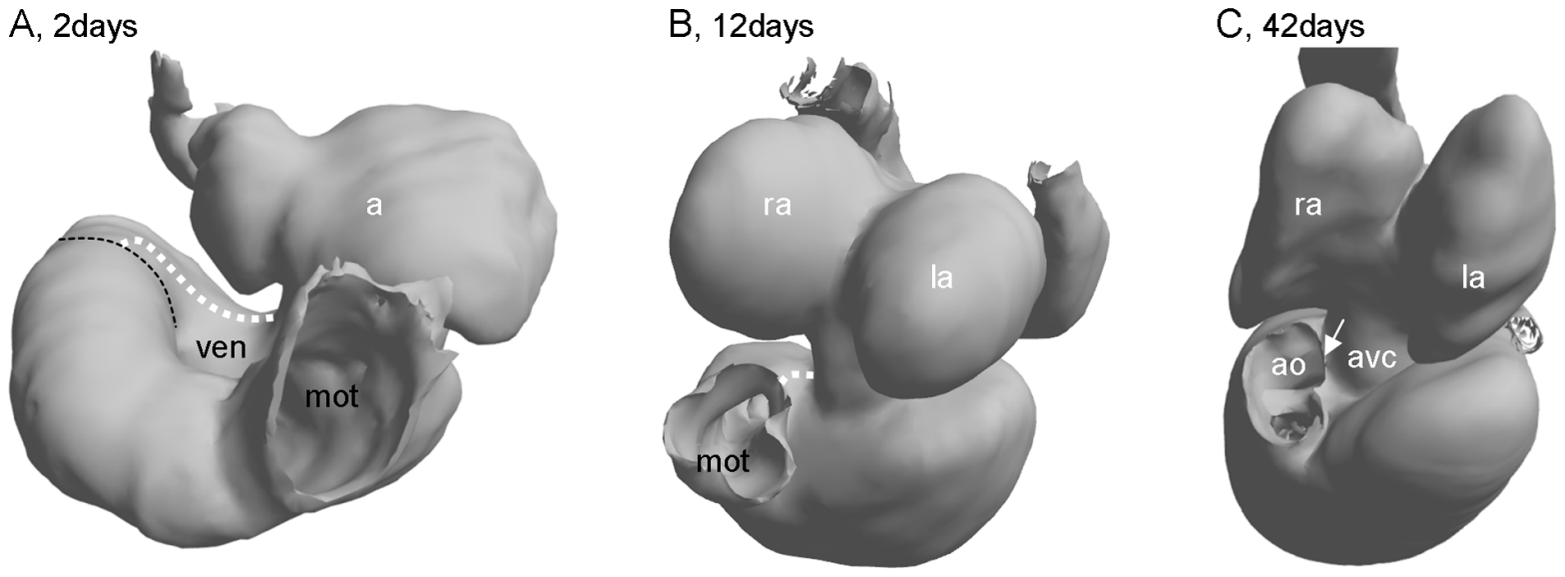
Ventricularization of the myocardial outflow tract of the corn snake. A. At 2 days the myocardial outflow tract (mot) constitutes most of the inner curvature of the heart (broken line) and the luminal length is 1180 µm. B. The myocardial outflow tract shortens to 470 µm by 12 days. C. By 42 days the myocardial outflow tract is shorter still (200 µm) and the inner curvature (arrow) is no more than a small piece of myocardium, the bulboauricularsporn of Greil [15], between the atrioventricular canal (avc) and the aortae (ao). a, atrium; la, left atrium; ra, right atrium.

At 2 days the myocardial outflow tract is thin-walled but nevertheless has little lumen because it is almost entirely filled with cushion material (the myocardial outflow tract may also be referred to as the conus arteriosus or the bulbus cordis). Its luminal length is more than 1000 µm as it curves from the extreme right of the ventricle to the left and then cranially (Fig. 7). By 10 days the conal length is halved to ca. 500 µm and from 35 days onwards it constitutes ca. 200 µm. Also by 10 days, at the distal end of the myocardial outflow tract, the single lumen is bifurcated by a mesenchymal protrusion into a (future) pulmonary channel and an aortic channel. Immediately distal hereof, the aortic channel is bifurcated by a second but smaller protrusion into a dorsal (future right aorta) and a ventral channel (future left aorta). The relative position of the three channels is maintained from 10 days on but the region of the bifurcation is further approximated to the ventricle as the myocardial outflow tract shortens. Concomitant with the ventricularization of the myocardial outflow tract, the myocardium between the atrioventricular canal and the aortic base shortens and thickens and has been coined the bulboauricularsporn by Greil [15] (Fig. 7).

## Discussion

### Cardiac development in squamates

Heart tube formation and looping has not been treated here and takes place prior to egg laying in the corn snake, the anole lizard and the Philippine sailfin lizard. These processes were described and visualized in the turtle *Chelydra serpentina*, where a straight heart tube is formed on the embryonic midline ca. 9 days post egg laying [38]. Looping has been described for the lizard *Lacerta agilis* [15] and the snake *Natrix natrix* [17], [18] (for heart development in crocodilians, see [39] and [40],[41]).

In using nomenclature of the fully formed heart on embryonic structures, it is important to appreciate that the formation of the heart is a highly dynamic process where many cells are added from extra-cardial precursor pools [42], [43]. Studies on cell fate and lineage tracing in chicken and mouse show that cells from the early outflow tract form the right ventricle and part of the ventricular septum, whereas the atrioventricular canal contributes to the entire left ventricular free wall [26], [30], [44], [45]. Amazingly, the myocardium of the early ventricle therefore contributes to little more than the left surface of the fully formed ventricular septum [30]. Similar studies remain to be made on reptiles.

The association of specific ventricular cava with an atrial inflow or an arterial outflow relates to distinct phases in formation of the ventricle; the early trabeculated ventricle, from ca. 2 days onward, is associated with the atrioventricular canal and gives rise to the cavum arteriosum and most of the cavum venosum. Only about 14 days later does the cavum pulmonale start to form by ventricularization of the myocardial outflow tract. Also, it is only by the ventricularization of the myocardial outflow tract that the aortic base becomes associated with the cavum venosum. A small bit of heart tube, the inner curvature of the ventricle, always remains between the right aortic base and the atrioventricular canal (the bulboauricularsporn of Greil, [15]). Thus, the position of the atrioventricular canal to the left of the right-sided myocardial outflow tract is maintained from the earliest stages of chamber formation to the fully formed heart.

### Chamber formation is similar in vertebrates

It has long been recognized that very early stages of reptilian cardiogenesis resemble other vertebrates with the formation of a heart tube that subsequently loops [12], [38], [41], [46], [47]. Subsequently, atrial and ventricular chamber formation proceeds as ballooning due to moderate-high proliferation on the outer curvature of the heart tube. The flanking myocardium, the smooth-walled inner curvature and myocardial outflow tract, remains heart tube-like, and is characterized by very low proliferation [48]–[51](Fig. S9). Such differentiation reflects an underlying molecular patterning that conveys differences in contractile and electrical properties and is governed by transcription factors [23], [52]–[55]. This concept of chamber growth opposes the segmental model, where chambers are thought to develop within distinct modules on the heart tube and the early heart is thought to contain all the precursors of the formed heart [43], [50], . On the contrary, cells from extracardiac precursor pools are being added to the heart at the venous and arterial poles and, amazingly, in mouse, the early chambered heart contains little more than the precursor for the left surface of the ventricular septum [30], [44]. The developmental program of mammals and birds, then, is distinguished by a great thickening of the compact wall of the ventricles [11], [50], [56].

### The sinus venosus is maintained in the formed hearts of ectotherms

The sinus venosus is arguably not a chamber as it does not form by ballooning [43], like the atria and ventricle, but we nonetheless categorize it as a chamber because it contains myocardium. Interestingly, the sinus venosus is retained throughout development as revealed with myocardial stains. Thus, the sinus venosus is much bigger than commonly perceived, namely the area where the three caval veins are confluent, and in fact includes the caval veins [5], [12], [14], [57]. Consistently, the ‘caval veins’ all contract prior to the atria (Movies S4-6) as also shown with electrocardiography [58], [59]. Also, in amphibians, the sinus venosus is an expanded cavity [60]–[64]. In fishes collectively, the sinus venosus can be fairly large, *e.g.* in hagfish, or may be much reduced, *e.g.* in zebrafish [65]–[67].

### Atrialization of venous myocardium does not occur in reptiles

Mammalian development sees incorporation (or atrialization) of the sinus venosus into the right atrium, which renders the dorsal wall smooth and the early atrium remains as the appendage characterized by pectinate/trabeculated musculature (*e.g.* [68]). The left atrium of mammals also acquires a smooth dorsal wall by incorporation of mediastinal, or pulmonary myocardium [68], [69]. The extent of atrialization of systemic and pulmonary venous myocardium varies a lot between mammalian species [70], [71]. It has been stated that parts of the sinus venosus is atrialized in reptiles [12] but the large size of this compartment and the absence of a smooth dorsal wall in the right atrium suggests otherwise. We never saw a myocardial sleeve around the pulmonary veins in the corn snake or in anole lizards. The left atrium remains trabeculated in its entirety.

### Comparative anatomy of the atria

**Figure 8:**
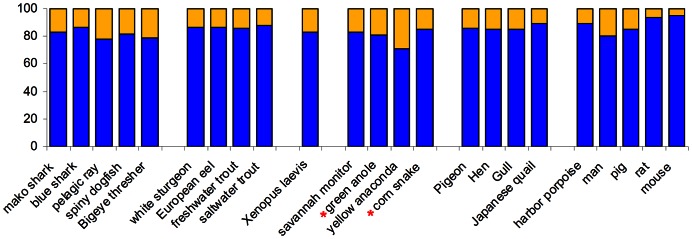
The relative masses of the atrium(atria) and ventricle(s) in vertebrates. Values based own data, the species studied here are marked by a red asterisk, or adopted from [92], [126]–[133].

The atrium, or atria, of ectothermic vertebrates is very voluminous compared to those of endotherms and contributes more to ventricular filling than in endotherms [10], [72]. Surprisingly, the atrium/atria of all vertebrates constitutes close to 20% of the ventricular mass despite the difference in importance for ventricular filling. There is a slight tendency for smaller atria in endotherms, but this could equally well be attributed to species-dependent variation (Fig. 8). At least in some fishes, there is substantial passive filling of the ventricle [73], [74].

The formed heart of all amniotes contains two atria separated by a complete septum, but only in mammals is the atrial septum formed by two septa. The two septa are the septum primum and, associated with the atrialization of the sinus venosus, the septum secundum. The septum primum is formed first and has a free edge towards the atrioventricular canal that expresses the transcription repressor Tbx3 [75], [76]. This expression pattern is also found in chicken and we have recently shown it to be conserved since reptiles [23]. We conclude that a septum secundum does not form in reptiles. Instead, in reptiles and birds the atrial septum has multiple perforations that are closed around the time of hatching [1], [77]. The most prominent trabeculation of the atria is the right atrial septum spurium (or suspensory ligament, [5], [14]), which is continuous with the left and right leaflets of the sinuatrial valve and can be found in back in sharks (the co-called dorsal commissure, [78]).

### The insulating plane is only found in endotherms

In embryogenesis, all vertebrates have a myocardial atrioventricular canal. Only in mammals and birds does the so-called insulating plane of fibro-fatty tissue ingress into the myocardium of the atrioventricular canal so that a single communication remains, the atrioventricular bundle [79]. Insulation between the atria and ventricle(s) is therefore primarily by the molecular patterning of the atrioventricular canal, and, secondarily, by the insulating plane [23], [55], [80]. Interestingly, in crocodiles only the ventral halve of the atrioventricular canal is interrupted by an insulating plane, whereas the dorsal halve remains myocardial. Crocodiles have a full ventricular septum and an atrioventricular valve apparatus like birds [81].

### The atrioventricular valve configuration is evolutionarily old

**Figure 9:**
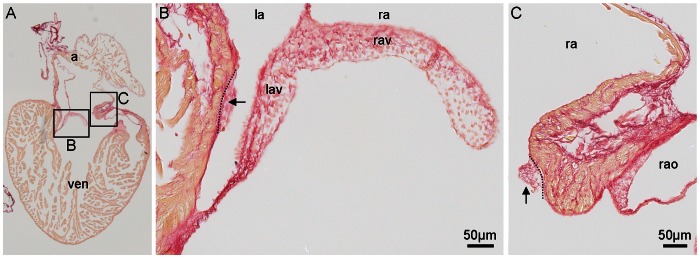
The atrioventricular valve complex of the formed lizard heart. In reptiles, the atrioventricular valve complex is dominated by large medial leaflets (B), albeit lateral cushions can be found (arrows, in B–C). 12 µm section of the adult heart of a green anole stained with picro-sirius red (collagen red, myocardium orange), a, atria; as, atrial septum; la, left atrium; lav, left atrioventricular valve leaflet; ra, right atrium; rav, right atrioventricular valve leaflet; ven, ventricle.

The mesenchyme of the atrioventricular canal is strikingly similar in amniotes with a large cushion dorsally and ventrally [1], [53]. This suggests that the fully formed left and right atrioventricular valve of the reptilian heart are homologous to the septal leaflet of the right-sided tricuspid valve and the aortic leaflet of the left-sided mitral valve of the mammalian heart. The reptilian heart also develops, to a variable degree, lateral cushions, again similar to other amniotes, and these may be retained in the formed heart and are most likely functionally redundant (Fig. 9). This configuration of four cushions further resembles the tetra-cuspid atrioventricular valve of many amphibians and fishes [1], [66], [82].

### Anatomy of the atrioventricular junction relates to septation

**Figure 10:**
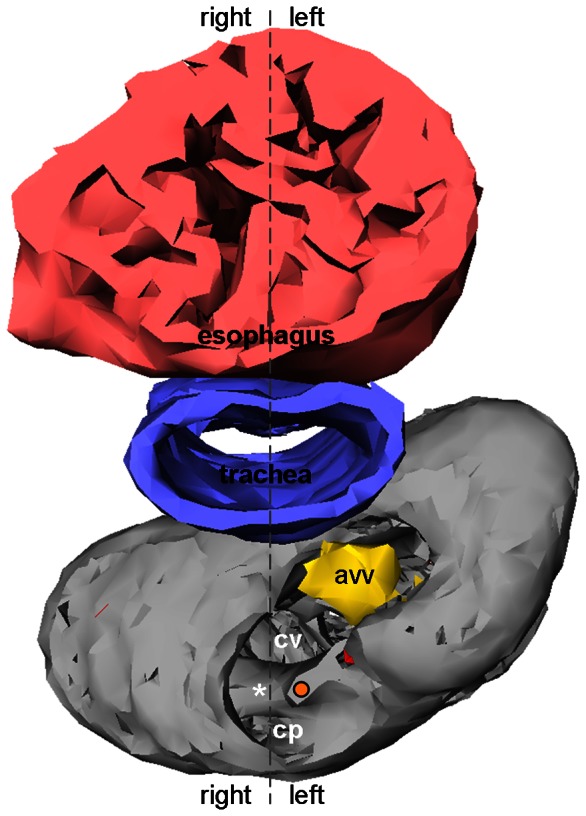
3D reconstruction of the ventricular base of near-hatching *Anolis sagrei* (st19). Using the esophagus and trachea to determine the body midline, it can be seen that the atrioventricular canal remains on the left side. asterisk, complete part of the muscular ridge; avv, atrioventricular valve; cp, cavum pulmonale; cv, cavum venosum; orange dot, free-standing part of the muscular ridge.

**Figure 11:**
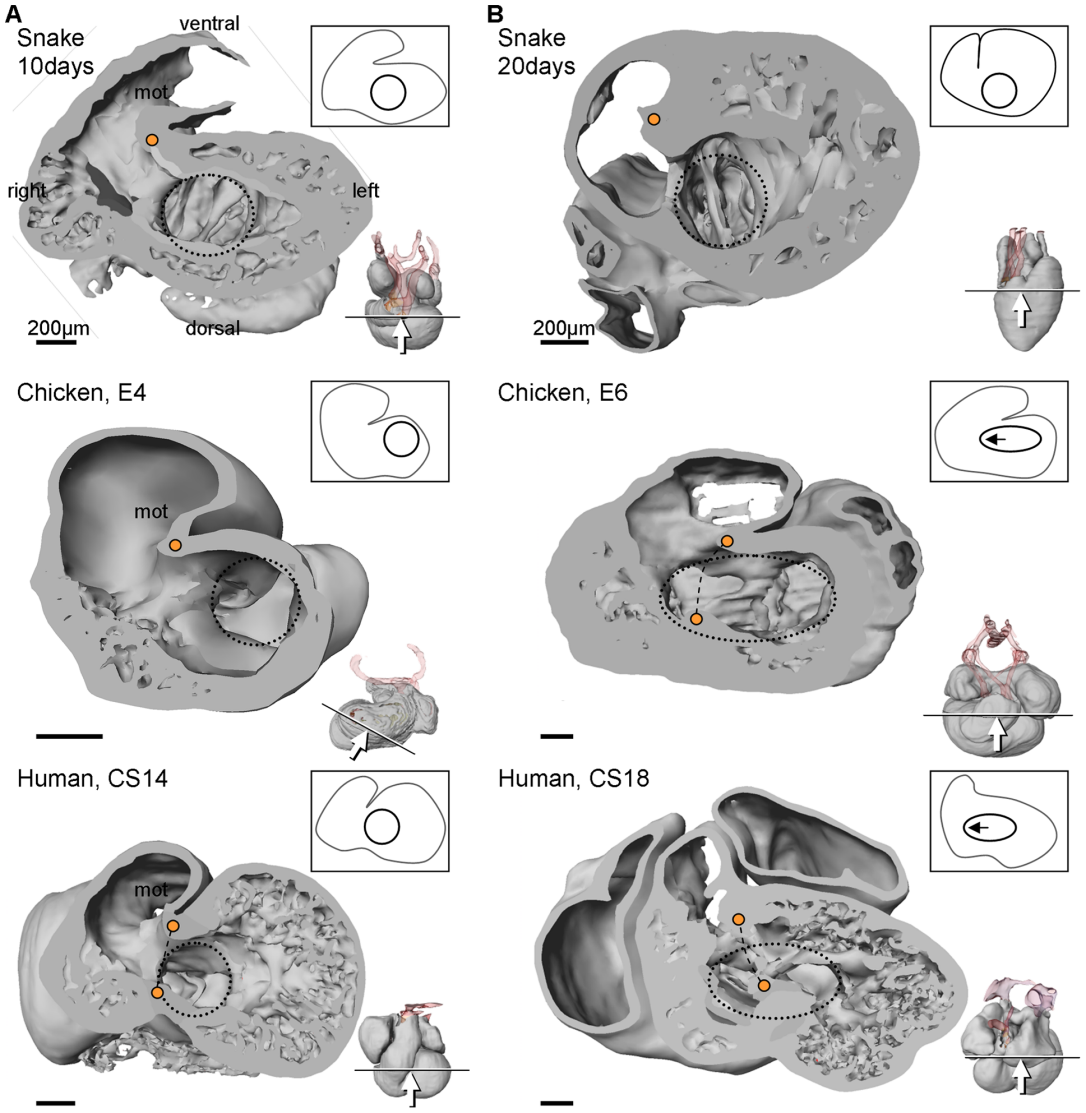
Comparative ventricular development. A. In early cardiac development, all amniote hearts show a bulbo-ventricular fold (orange dot) on the border of the trabeculated ventricle and the myocardial outflow tract. The atrioventricular canal (blue circle) is exclusively to the left of the fold. B. In non-crocodilian reptiles, the early design is maintained in later development. In amniotes with full ventricular septation (indicated by broken line between two orange dots) the atrioventricular canal expands to the right (note that compared to the stages in A, stages E6 and CS18 are ca. 5% further in gestation, whereas 20 days is ca. 15% further). Miniatures show sectioning plane and angle of inspection. CS, Carnegie stage; E, embryonic day.

**Figure 12:**
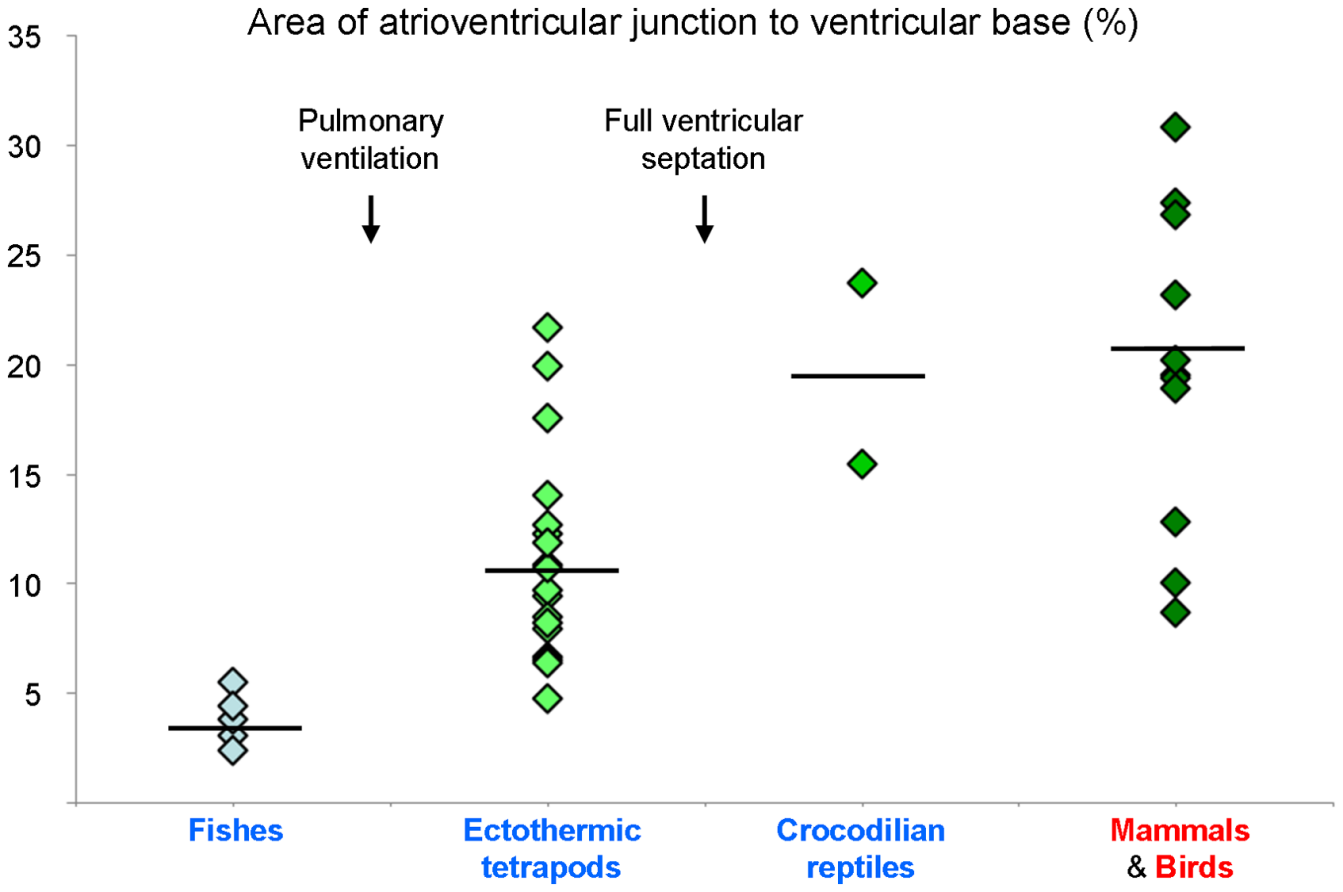
Size of the atrioventricular junction in vertebrates. Values are the cross sectional areas of the atrioventricular orifice(s) to the ventricular base (in %) measured on published and unpublished images in ImageJ (1.44d) using the ‘Polygon selections’ tool. In most cases, the state of contraction was not known. Horizontal bars are averages. In fishes, only systemic venous blood returns to the heart and the atrioventricular junction is small. With pulmonary ventilation, the heart receives both systemic and pulmonary venous blood (ectothermic tetrapods) and the atrioventricular junction more than doubles in size. In animals with full ventricular septation the atrioventricular junction is much larger (crocodilians, birds and mammals) regardless of ectothermic or endothermic metabolism. Published images were from [14], [15], [70], [71], [87], [112], [134]–[143].

Once the squamate atrioventricular canal has settled on the left of the body midline, it remains there throughout development (Fig. 10). It does not undergo the right-ward expansion and shift that occurs in animals with full ventricular septation (crocodilians, birds and mammals; Fig. 11) [15], [83], [84]. We therefore measured the size of the atrioventricular junction in formed hearts of vertebrates from published images and our own material; defined as the cross sectional area of the atrioventricular orifice(s) relative to the cross sectional area of the ventricular base, the atrioventricular junction constitutes less than 5% in fishes which have systemic circulation only, it is ca. 10% in ectothermic vertebrates which have both systemic and pulmonary circulation, and ca. 20% in the animals with full ventricular septation (Fig. 12).

### Ventricular trabeculations may not relate to septation

The earliest ventricular trabeculations in reptiles are aggregated into parallel sheets and a similar design can be seen in embryonic hearts of all vertebrates and in formed hearts of most fish and amphibians and reptiles [17], [22], [53], [70], [72], [85]–[88]. The vertical septum of the formed non-crocodilian ventricle is among these sheets and because it is positioned immediately caudal to the atrioventricular valves, it has repeatedly been hypothesized to constitute an important part of the complete ventricular septum of mammals and birds (cf. [3]). It is after the initiation of septation, however, that the center of the mammalian and avian atrioventricular canal is positioned immediately above the forming septum by expansion and shift to the right (Fig. 11). Also, in mammals and birds, the crest of the ventricular septum expresses *Bmp2* and *Tbx3* and a similar crest has not been found in lizards [23]. Furthermore, the ventricular septum has a left-right gradient of Tbx5 expression [89]. In reptiles, the cranial part of the cavum pulmonale does not express *Tbx5* and the left-right gradient is thus established over the muscular ridge, rather than the vertical septum (Fig. 1A–B, [4]). It is thus arguable that the vertical septum does not relate to the ventricular septum.

### The bulboauricularlamella are a common feature of vertebrate ventricles

**Figure 13:**
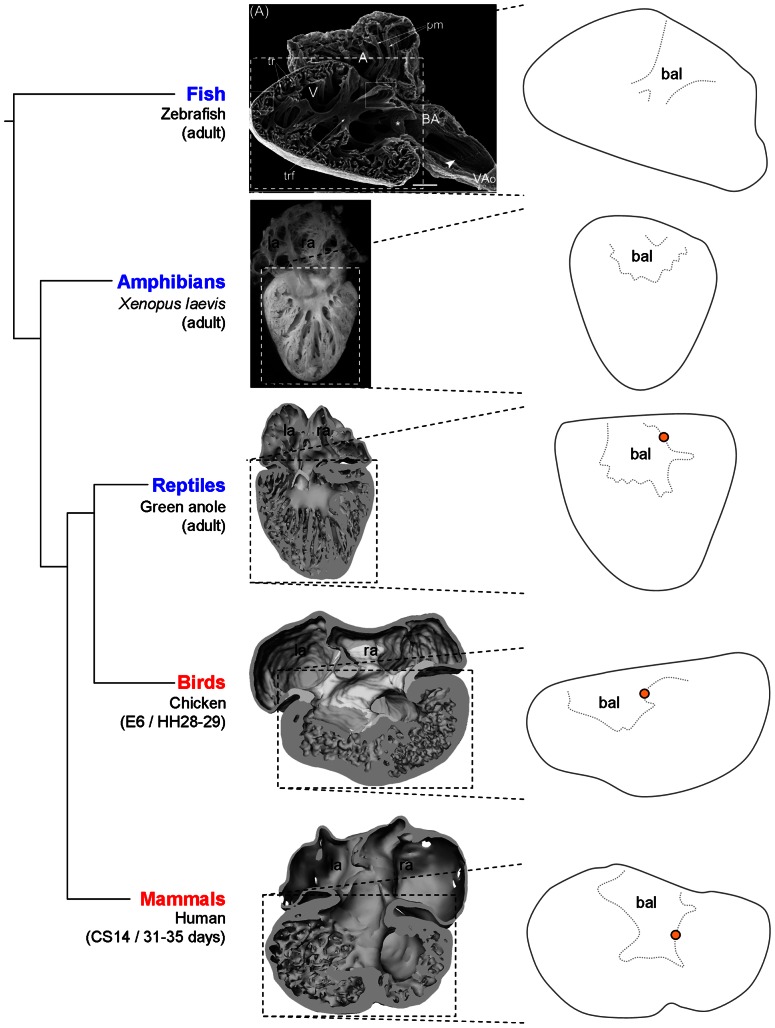
The bulboauricularlamella are a feature of most vertebrate hearts. In most adult ectotherms smooth-surfaced myocardium, the bulboauricularlamelle (bal), is interspersed between the atrioventricular canal and the ventricular trabeculations. A similar design is seen in embryonic hearts of mammals and birds. orange dot, bulboventricular fold or free-standing part of the muscular ridge. The image of the zebrafish heart is adopted and modified from [65].

The atrioventricular canal is initially a free standing tube, but becomes enclosed by ventricular myocardium and acquires a funnel-like appearance (it is also known as the atrioventricular funnel or ‘Trichter’ in German). In reptiles, the atrioventricular canal myocardium is contiguous with smooth-surfaced ventricular myocardium referred to as bulboauricularlamella [15]. The bulboauricularlamella can also be recognized in the embryonic hearts of mammals and birds as ‘gullies’ that contribute to atrioventricular valve formation (Fig. 13) [84], [90], [91]. Fishes and amphibians, also develop bulboauricularlamella [70]. In mouse it has been shown that the atrioventricular myocardium contributes much to the left ventricle [30] and we speculate that the bulboauricularlamella constitute traces of this process.

### Only in amniotes is the myocardial outflow tract ventricularized

In contrast to amniotes, the myocardial outflow tract of fishes and amphibians is retained in the adult formed heart. It is prominent in cartilaginous fish, where it constitutes ca. 10–20% of the entire cardiac mass, in sarcopterygii fish like lungfishes, and basal actinopterygii fish, such as sturgeons as well as most amphibians [66], [92]–[95]. In fishes in general, the myocardial outflow tract contains one to eight rows of valves in the transverse plane, but only in lungfishes have the multiple valves fused to two longitudinal ridges [1], [66], [96], [97], [97]–[101]. In amphibians, a spiral valve of connective tissue may divide the myocardial outflow tract into a systemic and a pulmocutaneous channel. The ventricularization of the myocardial outflow tract is thus a defining event in amniotes [102] and the developmental regression, ca. 1 mm in length, has also been measured in man [103], [104] and chicken [26]. It has long been held that the myocardial outflow tract contributes by ventricularization to the full ventricular septum in mammals, birds and crocodilies [1], [3], [12], [15], [105]–[109]. Yet, to the best of our knowledge there is no explanation as to why the myocardial outflow tract ventricularizes in reptiles, representing the ancestral amniote condition. In the derived condition of mammals and birds, the ventricularization essentially creates the right ventricle and is probably driven by push from cells being added distally to the outflow tract from extracardiac precursors [26], [42], [44], [51]. In zebrafish development, cells are also added to the arterial pole, but the myocardial outflow myocardium never grows to the proportion of the heart seen in amniotes (cf. the miniatures in Fig. 1) and a septation like the muscular ridge is not manifested [110], [111].

### Structures derived from the ventricularized myocardial outflow tract

In reptiles, the myocardial outflow tract is partially divided by a muscular protrusion, which is continuous with the muscular ridge caudally and the arterial aorticopulmonary septum cranially. Therefore, as the muscular outflow tract becomes ventricularized this protrusion blends seamlessly into the muscular ridge and is considered a part of it. It has been suggested that the protrusion is homologous to the spiral valve of the amphibian heart and the so-called conal septum of the mammalian heart that separates the lumen of the right ventricular outflow tract from the aorta [3], [83]. Also, the complete part and the free-standing part of the muscular ridge is said to resemble the trabecula septomarginalis (incomplete part) and moderator band (complete part) of the mammalian and avian right ventricle [15], [21], [70], [105], [107], [112]. Consistently, a small remnant of the myocardial outflow tract can be found ventrally in the reptile heart, at the pulmonary arterial base, like the right ventricular outflow tract (or conus, or infundibulum) of the mammalian right ventricle [71], [113]–[116]. It remains an intriguing question how the heart tube-like outflow tract myocardium acquires a ventricular phenotype upon being incorporated into the ventricle.

### The bulbuslamelle may contribute to the right atrioventricular valve complex

**Figure 14:**
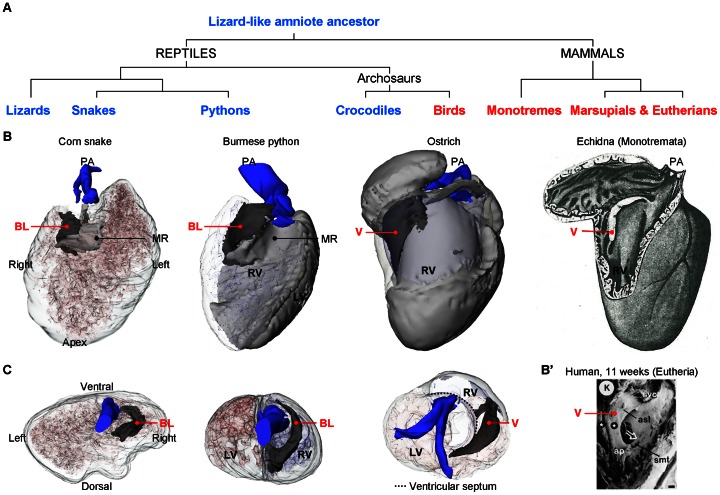
Evolutionary fates of the bulbuslamelle. **A**. Phylogenetic tree to show the show the evolution of selected groups of amniotes. **B**. Ventricles inspected from the right with the ventricular wall made transparent so that the similarity in design between the bulbuslamelle (BL) and the myocardial right atrioventricular valve (V) can be appreciated. The V of birds and monotreme mammals is positioned in the heart where the bulbuslamelle (BL) of the reptile heart is situated. In pythons, the BL is strongly developed and participates in separating the left and right sides of the ventricle. The image of the echidna heart is modified from [70]. **B'**. Scanning electron microscopic image of the human heart at 11 weeks of development with the wall of the right ventricle removed (modified from [90]). In the human heart, and eutherian mammals in general, the anterosuperior leaflet (asl) of the tricuspid valve is muscular until late in development. **C**. Cranial view of the ventricular base showing the position of the BL in the ‘basal’ condition (corn snake), in the contributing to separate the left and right sides of the python ventricle and as atrioventricular valve (ostrich) (the ventricle of the corn snake was deformed during fixation, as seen by indentations on the dorsal surface). ap, anterior papillary muscle; smt, septomarginal trabeculation; svc, supraventricular crest; stars, tricuspid gully complex. PA, pulmonary artery.

It is perplexing that birds and monotreme mammals have a mural muscular flap valve at the right atrioventricular junction, because they evolved independently from reptilian ancestors where a similar structure is not easily recognized (Fig. 14) [46], [70], [117]–[120]. Only crocodiles also have a right muscular flap valve and they are grouped as archosaurs together with dinosaurs and birds [121]–[123]. Marsupial and eutherian mammals do not have a right muscular flap valve, yet, in atrioventricular valve development, the valvular complexes are partly muscular [90], [91]. And persisting muscularity of the valve may lead to the congenital malformation of Ebstein's anomaly [84], [124]. Common to crocodiles, birds and mammals is a full ventricular septation, which, as shown in Figure 9, is associated with a right-ward expansion of the atrioventricular canal. The right margin of the atrioventricular canal then approaches the position of the bulbuslamelle as found in the reptile heart and the bulbuslamelle may therefore contribute to the formation of the myocardial right atrioventricular valve (Figs. 1, 9, 11) [22], [84].

## Summary

The developing reptile heart is characterized by fast growth of the chambers and slow growth of the flanking segments, exactly like in mammals and birds. In reptiles, a myocardial sinus venosus is retained in the formed heart, whereas the sinus venosus of mammals atrializes to the right atrium to form a vestibule in the dorsal right atrial wall. Associated herewith, is the absence (reptiles) and presence (mammals) of an atrial septum secundum. A myocardial sleeve is absent from the reptilian pulmonary vein and there is no vestibule in the left atrium. The atrioventricular canal of reptiles is not interrupted by an insulating plane, as in mammals and birds. Also, the atrioventricular canal remains on the cardiac left, whereas it expands and migrates to the right in mammals and birds (and crocodilians) associated with the formation of the ventricular septum.

## Glossary

aortico-pulmonary septum: the arterial septum of the truncus arteriosus that separates the pulmonary arterial lumen from the aortic lumina.

bulboauricularlamelle, dorsal: smooth-surfaced ventricular myocardium, situated in the inner-most dorsal base of the ventricle in between the atrioventricular canal and the arteries.

bulboauricularlamelle, ventral: smooth-surfaced ventricular myocardium, situated in the inner-most ventral base of the ventricle in between the atrioventricular canal and the arteries.

bulboauricularsporn: thickened myocardium in between the atrioventricular canal and the right aorta. Derived from the inner curvature of the embryonic heart tube. Synonyms; bolvo ventricular spur [125], conoauricular flange [103].

bulbo-ventricular fold: the inner curvature of the transition from ventricle to myocardial outflow tract. Synonyms; bolvo ventricular spur, conoventicular flange [103].

bulbuslamelle: smooth-surfaced ventricular myocardium juxtaposed to the muscular ridge. In pythons and varanid lizards, it separates the cavum pulmonale and the cavum venosum during ventricular systole. Defined here as the smooth-surfaced structure to the right of the bulboauricularsporn, and therefore the atrioventricular canal, that holds the dorsal valves of the aortae. Derived from the ventricularization of the myocardial outflow tract. Synonyms; bulbar flange.

cardiac shunt, left-to-right: the circulation of pulmonary venous blood (oxygen-rich) to the pulmonary circulation.

cardiac shunt, right-to-left: the circulation of systemic venous blood (oxygen-poor) to the systemic circulation.

cavum arteriosum: the left-sided chamber of the ventricle that receives blood (oxygen-rich) from the left atrium; *i.e.* the ‘systemic side’ of the ventricle.

cavum dorsale: the combined cavum arteriosum and cavum venosum, *i.e.* the cavities to the left of the muscular ridge and the bulbuslamelle.

cavum pulmonale: the right-sided chamber of the ventricle that receives blood (oxygen-poor) from the right atrium in diastole; *i.e.* part of the ‘pulmonary side’ of the ventricle. Synonyms; cavum ventrale.

cavum venosum: the central chamber of the ventricle siyuated to the right of the vertical septum and to the left of the muscular ridge and the bulbuslamelle. It receives (oxygen-poor) blood from the right atrium in diastole and is traversed by blood (oxygen-rich) from cavum arteriosum in systole; *i.e.* part of the ‘pulmonary side’ of the ventricle.

cavum ventrale: synonym for the cavum pulmonale.

muscular ridge: the smooth-surfaced myocardial ridge-like structure of the ventricle to the right of the atrioventricular canal that is continuous with the aortico-pulmonary septum and holds the ventral valves of the aortae and the dorsal valve of the pulmonary artery. Synonyms; horizontal septum, Muskelleiste, cloison helïcoidale, ventricular septum, septum interventriculare (a detailed literature review was made by Webb *et al*., 1974).

myocardial outflow tract: heart tube myocardium downstream of the ventricle. Synonyms; conus arteriosus, bulbus arteriosus, conus cordis, bulbus cordis.

septum spurium: prominent trabeculation of the roof of the right atrium associated with the sinuatrial valves. Synonyms; suspensory ligament, dorsal commissure.

spannmuskel: trabecular sheets in the ventricle that are caudal to the bulbuslamelle and continuous with it.

truncus arteriosus: the arterial trunk proximal to the ventricle containing the pulmonary artery and the aortae. Synonyms; bulbus arteriosus.

vertical septum: a sheet (or sheets according to some authors) of spongy myocardium in the ventricle situated immediately under the atrioventricular canal. The atrioventricular valves ‘plunge’ unto it during diastole.

## Supporting Information

Figure S1 **3D models of the heart of the corn snake (** ***Pantherophis guttatus*** **), 2 to 16** **days post laying.**
(PDF)

Figure S2 **3D models of the heart of the corn snake (** ***Pantherophis guttatus*** **), 20** **days post laying to 3** **months.**
(PDF)

Figure S3 **3D models of the heart of the anole lizard.**
(PDF)

Figure S4 **3D models of the heart of the embryonic chicken (4 and 6** **days post laying).**
(PDF)

Figure S5 **3D models of the heart of embryonic man (Carnegie stages 14 and 18).**
(PDF)

Figure S6 **3D model of the heart of the adult Burmese python (** ***Python molurus*** **).**
(PDF)

Figure S7 **3D model of the heart of the adult ostrich (** ***Struthio camelus*** **).**
(PDF)

Figure S8 **Growth of cardiac compartments**. The atrial and ventricular compartments showed exponential growth, whereas there was little change in the myocardial volume of the atrioventricular canal and the myocardial outflow tract.
(TIF)

Figure S9 **Proliferation of the hearts in two specimens of anole lizard, as assessed by BrdU incorporation.** **A**. A 7 µm section of the st5 specimen, close to the transverse plane, showing myocardium (blue), nuclei (green) and BrdU positive nuclei (orange). **B**. A 7 µm section of the st12 specimen, close to the horizontal plane, showing myocardium (blue), nuclei (green) and BrdU positive nuclei (orange). **C**–**D**. Reconstructions of the myocardium upon which is projected the fraction of BrdU positive nuclei as described in [50]. The color-scale bar indicates BrdU incorporation in zero (0) to every fourth nuclei (0.25). **E**. Internal view of the ventral halve of the st5 specimen showing relatively high proliferation in the ballooning atria (la, left atrium; ra, right atrium) and ventricle (ven). **F**. Internal view of the ventral halve of the st12 specimen. At this stage the cardiac compartments almost have the proportions of the fully formed heart and proliferation is lower than in the st5 specimen, albeit the outer curvature of the ventricle still shows some proliferation.
(TIF)

Movie S1 **Seen from the right, the beating heart of a corn snake embryo 10** **days after egg laying.** mot, myocardial outflow tract.
(AVI)

Movie S2 **Seen ventrally, the beating heart of a corn snake embryo 10** **days after egg laying.** mot, myocardial outflow tract.
(AVI)

Movie S3 **Seen from the left, the beating heart of a corn snake embryo 10** **days after egg laying.** mot, myocardial outflow tract.
(AVI)

Movie S4 **Seen from the right, the beating heart of a corn snake embryo 20** **days after egg laying.** mot, myocardial outflow tract.
(AVI)

Movie S5 **Seen ventrally, the beating heart of a corn snake embryo 20** **days after egg laying.** mot, myocardial outflow tract.
(AVI)

Movie S6 **Seen from the left, the beating heart of a corn snake embryo 20** **days after egg laying.** mot, myocardial outflow tract.
(AVI)
