# Supplementary material for: Development of the Hearts of Lizards and Snakes and Perspectives to Cardiac Evolution
Source: PLoS One. 2013 Jun 5;8(6):e63651. doi: 10.1371/journal.pone.0063651 (PMC3673951; doi:10.1371/journal.pone.0063651)
Supplement: Figure S1 — 3D models of the heart of the corn snake ( Pantherophis guttatus ), 2 to 16 days post laying. (PDF) [file pone.0063651.s001.pdf]

Heart of the corn snake, 2 dpl

- lumen
- cushions
- sinus venosus
- atrium
- atrioventricular canal
- ventricle
- bulboventricular fold
- conus

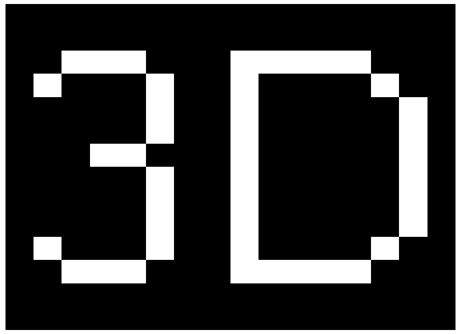

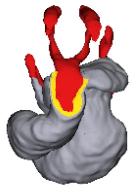

Ventral

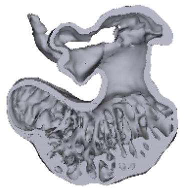

Fig. 4A

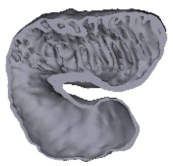

Fig. 5

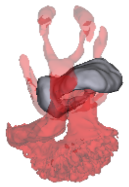

Atrium

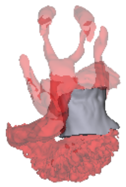

AVC

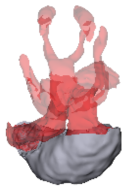

Ventricle

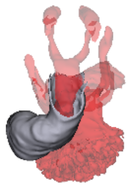

MOT

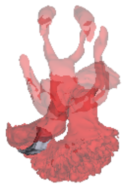

Bulboventricular fold

Heart Failure
Research Center
http://3d.hfrc.nl

Heart of the corn snake, 10 dpl

- lumen
- pulmonary vein
- cushions
- sinus venosus
- sinuatrial valves
- atria
- septum spurium
- atrioventricular canal
- ventricle
- bulboauricularlamella
- muscular ridge
- bulbuslamelle
- conus

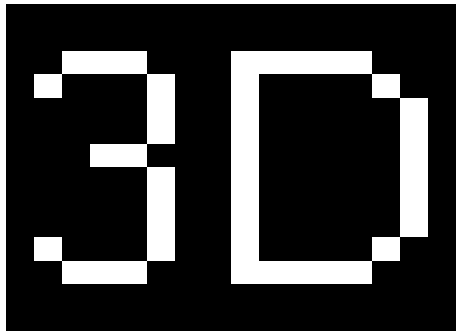

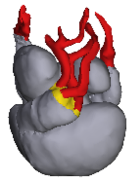
Ventral

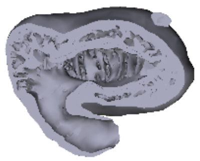
Fig. 5

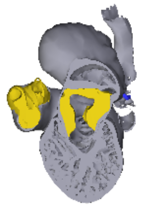
Fig. 6A

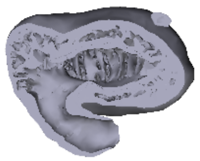
Fig. 6A′

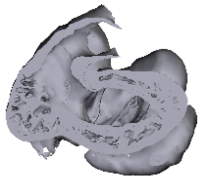
Fig. 11

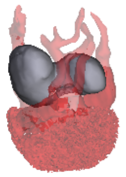
Atria

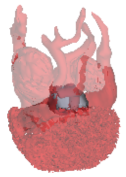
AVC

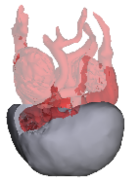
Ventricle

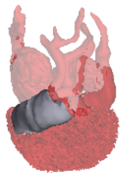
MOT

Heart Failure
Research Center
http://3d.hfrc.nl

Heart of the corn snake, 12 dpl

- lumen
- pulmonary vein
- cushions
- sinus venosus
- sinuatrial valves
- atria
- septum spurium
- atrioventricular canal
- ventricle
- bulboauricularlamella
- muscular ridge
- bulbuslamelle
- conus

3D

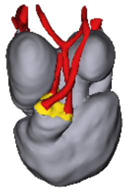
Ventral

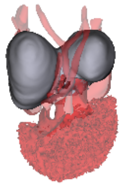
Atria

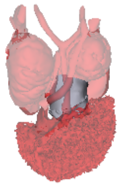
AVC

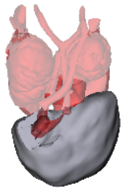
Ventricle

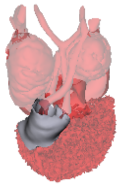
MOT

Heart Failure
Research Center
http://3d.hfrc.nl

Heart of the corn snake, 14 dpl

- lumen
- pulmonary vein
- cushions
- sinus venosus
- sinuatrial valves
- atria
- septum spurium
- atrioventricular canal
- ventricle
- bulboauricularlamella
- muscular ridge
- bulbuslamelle
- conus

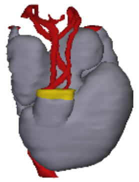
Ventral

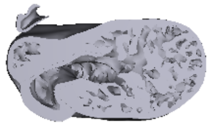
Fig. 5

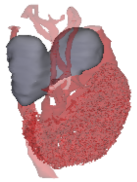
Atria

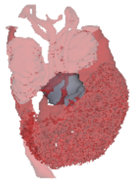
AVC

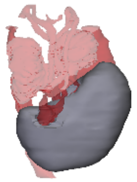
Ventricle

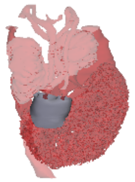
MOT

Heart Failure
Research Center
http://3d.hfrc.nl

Heart of the corn snake, 16 dpl

- lumen
- pulmonary vein
- cushions
- sinus venosus
- sinuatrial valves
- atria
- septum spurium
- atrioventricular canal
- ventricle
- bulboauricularlamella
- muscular ridge
- bulbuslamelle
- conus

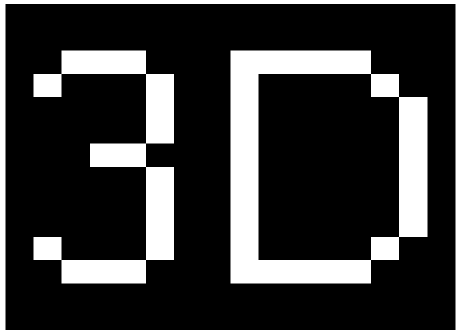

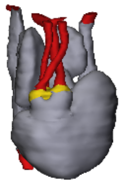
Ventral

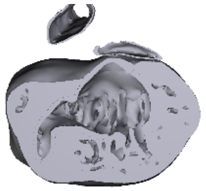
Fig. 5

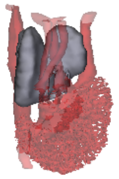
Atria

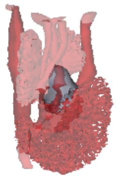
AVC

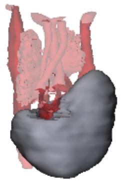
Ventricle

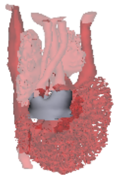
MOT

Heart Failure
Research Center
http://3d.hfrc.nl
