# Supplementary material for: Development of the Hearts of Lizards and Snakes and Perspectives to Cardiac Evolution
Source: PLoS One. 2013 Jun 5;8(6):e63651. doi: 10.1371/journal.pone.0063651 (PMC3673951; doi:10.1371/journal.pone.0063651)
Supplement: Figure S2 — 3D models of the heart of the corn snake ( Pantherophis guttatus ), 20 days post laying to 3 months. (PDF) [file pone.0063651.s002.pdf]

Heart of the corn snake, 20 dpl

- lumen
- pulmonary vein
- cushions
- sinus venosus
- sinuatrial valves
- atria
- septum spurium
- atrioventricular canal
- ventricle
- bulboauricularlamella
- vertical septum
- muscular ridge
- bulbuslamelle
- conus

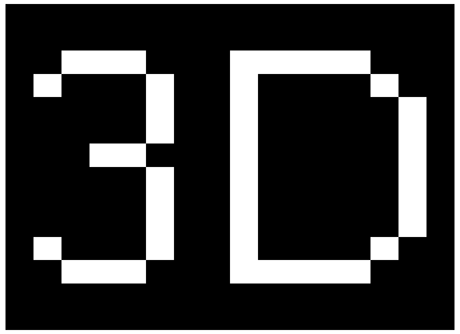

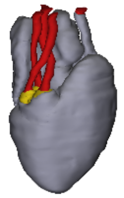

Ventral

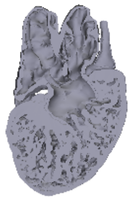

Fig. 4A

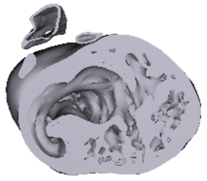

Fig. 5

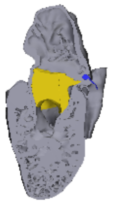

Fig. 6B

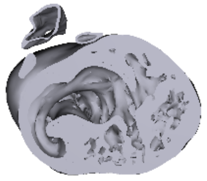

Fig. 6B’

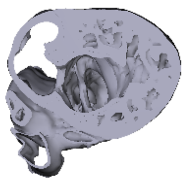

Fig. 11

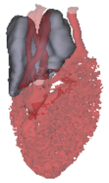

Atria

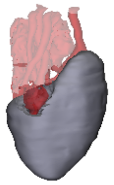

Ventricle

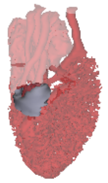

MOT

Heart Failure
Research Center
http://3d.hfrc.nl

Heart of the corn snake, 26 dpl

- lumen
- pulmonary vein
- cushions
- sinus venosus
- sinuatrial valves
- atria
- septum spurium
- atrioventricular canal
- ventricle
- bulboauricularlamella
- vertical septum
- muscular ridge
- bulbuslamelle
- conus

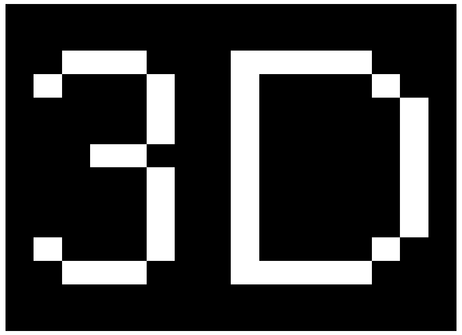

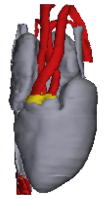
Ventral

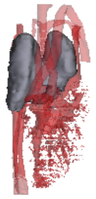
Atria

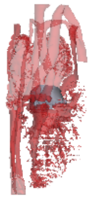
AVC

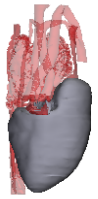
Ventricle

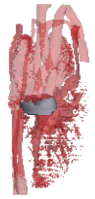
MOT

Heart Failure
Research Center
http://3d.hfrc.nl

Heart of the corn snake, 35 dpl

- lumen
- pulmonary vein
- cushions
- sinus venosus
- sinuatrial valves
- atria
- septum spurium
- atrioventricular canal
- ventricle
- bulboauricularlamella
- vertical septum
- muscular ridge
- bulbuslamelle
- conus

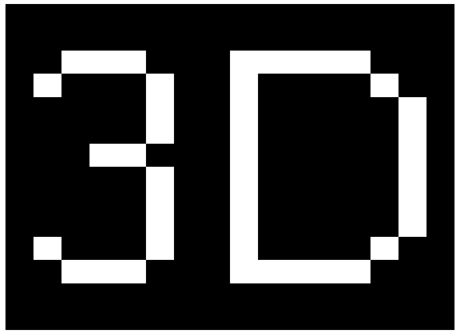

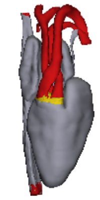
Ventral

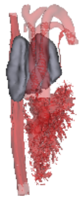
Atria

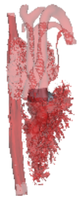
AVC

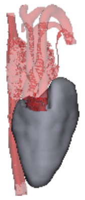
Ventricle

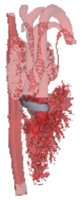
MOT

Heart Failure
Research Center
http://3d.hfrc.nl

Heart of the corn snake, 42 dpl

lumen
pulmonary vein
cushions
sinus venosus
sinuatrial valves
atria
septum spurium
atrioventricular canal
ventricle
bulboauricularlamella
vertical septum
muscular ridge
bulbuslamelle
conus

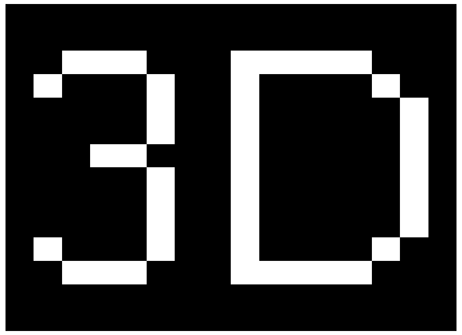

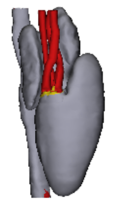

Ventral

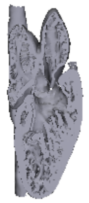

Fig. 4A

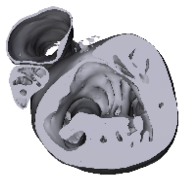

Fig. 5

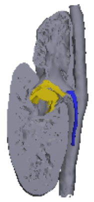

Fig. 6C

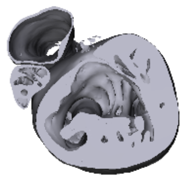

Fig. 6C′

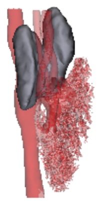

Atria

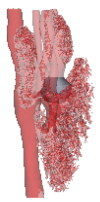

AVC

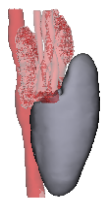

Ventricle

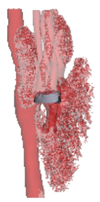

MOT

Heart Failure
Research Center
http://3d.hfrc.nl

Heart of the corn snake, 3 m

- lumen
- pulmonary vein
- cushions
- sinus venosus
- sinuatrial valves
- atria
- septum spurium
- atrioventricular canal
- ventricle
- bulboauricularlamella
- vertical septum
- muscular ridge
- bulbuslamelle
- conus

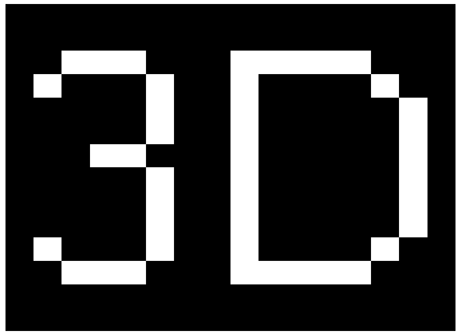

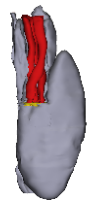
Ventral

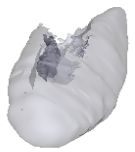
Fig. 14B

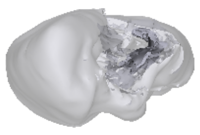
Fig. 14C

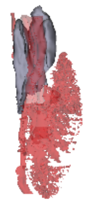
Atria

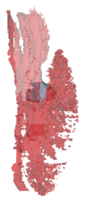
AVC

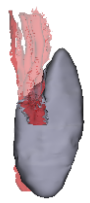
Ventricle

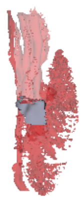
MOT

Heart Failure
Research Center
http://3d.hfrc.nl
