# Supplementary material for: Development of the Hearts of Lizards and Snakes and Perspectives to Cardiac Evolution
Source: PLoS One. 2013 Jun 5;8(6):e63651. doi: 10.1371/journal.pone.0063651 (PMC3673951; doi:10.1371/journal.pone.0063651)
Supplement: Figure S3 — 3D models of the heart of the anole lizard. (PDF) [file pone.0063651.s003.pdf]

Heart of the anole lizard, Sanger stage 5

- lumen
- sinus venosus
- atrium
- atrioventricular canal
- ventricle
- bulboventricular fold
- conus

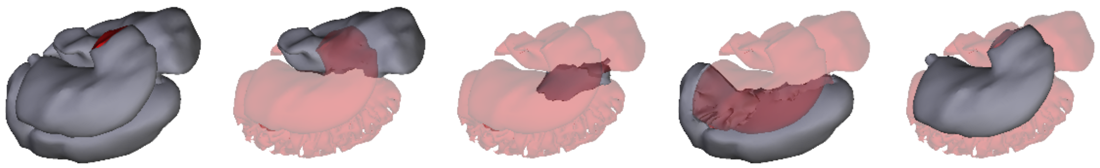

Ventral | Atrium | AVC | Ventricle | MOT

Heart Failure
Research Center
http://3d.hfrc.nl

Heart of the anole lizard, Sanger stage 9

- lumen
- mesenchyme
- sinus venosus
- right atrium
- left atrium
- atrial septum
- atrioventricular canal
- ventricle
- conus

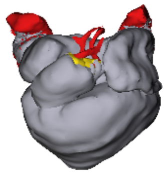
Ventral

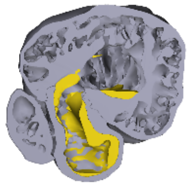
Fig. 1D

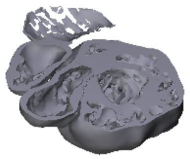
Fig. 1F

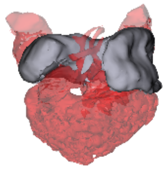
Atria

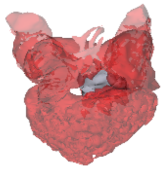
AVC

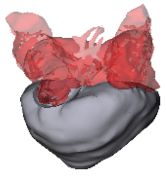
Ventricle

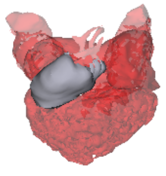
MOT

Heart Failure
Research Center
http://3d.hfrc.nl

Heart of the anole lizard, Sanger stage 17

- lumen
- atrioventricular valves
- sinus venosus
- atria
- atrioventricular canal
- ventricle
- bulboauricularlamella
- vertical septum
- muscular ridge
- bulbuslamelle
- conus
- truncus arteriosus

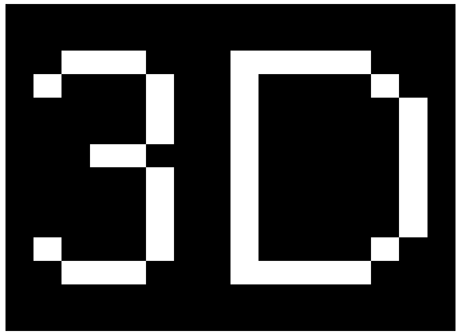

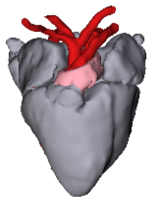

Ventral

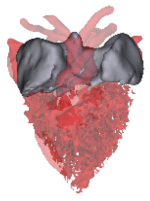

Atria

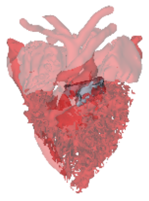

AVC

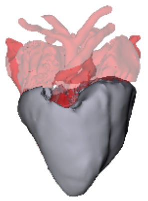

Ventricle

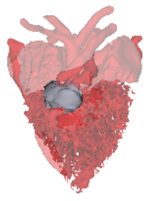

MOT

Heart Failure
Research Center
http://3d.hfrc.nl

Heart of the adult anole lizard

- lumen of the left atrium
- lumen of cavum arteriosum
- right aorta
- left aorta
- lumen of cavum venosum
- lumen of the right atrium
- lumen of cavum pulmonale
- pulmonary artery
- atrioventricular valves
- lateral atrioventricular valves
- arterial valves
- cartilago cordis
- collagen
- left atrium
- right atrium
- atrial spetum
- atrioventricular canal
- dorsal bulboauricularlamelle
- ventral bulboauricularlamelle
- vertical septum
- muscular ridge
- bulbuslamelle
- Spannmuskel of Greil
- cavum arteriosum
- cavum venosum
- cavum pulmonale
- conus arteriosus
- truncus arteriosus

Heart Failure
Research Center
http://3d.hfrc.nl

3D

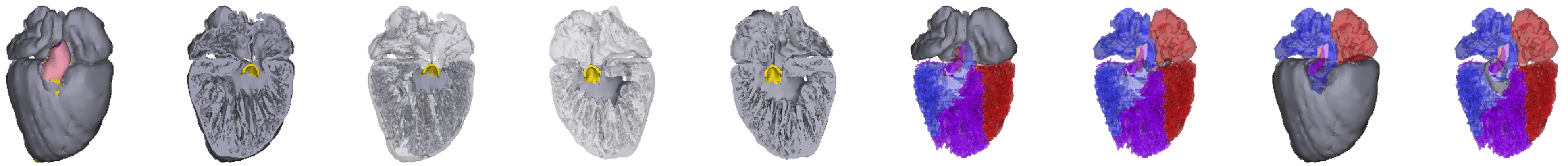

Ventral | Fig. 6D | Fig. 6D′ | Fig. 6E | Fig. 13 | Atria | AVC | Ventricle | MOT
