# Supplementary material for: Development of the Hearts of Lizards and Snakes and Perspectives to Cardiac Evolution
Source: PLoS One. 2013 Jun 5;8(6):e63651. doi: 10.1371/journal.pone.0063651 (PMC3673951; doi:10.1371/journal.pone.0063651)
Supplement: Figure S4 — 3D models of the heart of the embryonic chicken (4 and 6 days post laying). (PDF) [file pone.0063651.s004.pdf]

# Heart of the chicken, 4 dpl

- lumen
- cardiac jelly
- sinus venosus
- atrium
- atrioventricular canal
- ventricle
- conus
- trachea
- esophagus

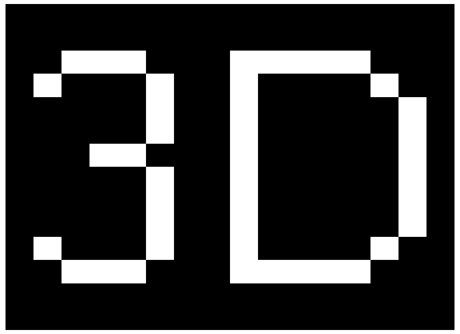


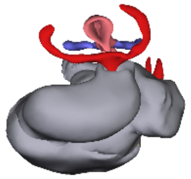
Ventral

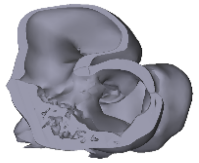
Fig. 11

Heart Failure
Research Center
http://3d.hfrc.nl

# Heart of the chicken, 6 dpl

- lumen
- mesenchyme
- myocardium
- trachea
- esophagus

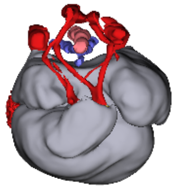

Ventral

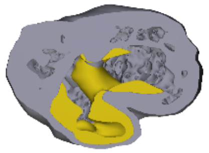

Fig. 1G

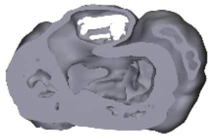

Fig. 11

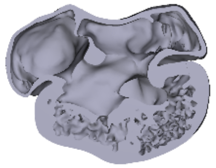

Fig. 13

Heart Failure
Research Center
http://3d.hfrc.nl
