# Supplementary material for: Development of the Hearts of Lizards and Snakes and Perspectives to Cardiac Evolution
Source: PLoS One. 2013 Jun 5;8(6):e63651. doi: 10.1371/journal.pone.0063651 (PMC3673951; doi:10.1371/journal.pone.0063651)
Supplement: Figure S5 — 3D models of the heart of embryonic man (Carnegie stages 14 and 18). (PDF) [file pone.0063651.s005.pdf]

Heart of man, Carnegie stage 14

lumen

mesenchyme

myocardium

3D

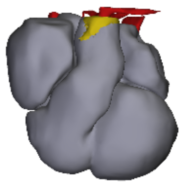

Ventral

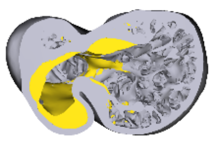

Fig. 1H

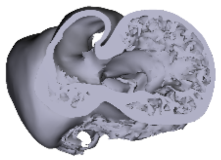

Fig. 11

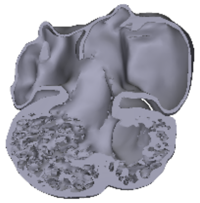

Fig. 13

Heart Failure
Research Center
http://3d.hfrc.nl

Heart of man, Carnegie stage 18

lumen

mesenchyme

myocardium

3D

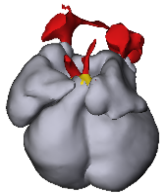

Ventral

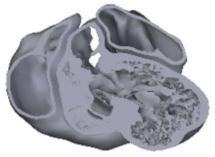

Fig. 11

Heart Failure
Research Center
http://3d.hfrc.nl
