# Supplementary material for: Development of the Hearts of Lizards and Snakes and Perspectives to Cardiac Evolution
Source: PLoS One. 2013 Jun 5;8(6):e63651. doi: 10.1371/journal.pone.0063651 (PMC3673951; doi:10.1371/journal.pone.0063651)
Supplement: Figure S6 — 3D model of the heart of the adult Burmese python ( Python molurus ). (PDF) [file pone.0063651.s006.pdf]

Heart of the adult Burmese python

- oxygen rich blood
- oxygen poor blood
- atrioventricular valves
- arterial valves
- atria
- ventricle
- bulbuslamelle
- truncus

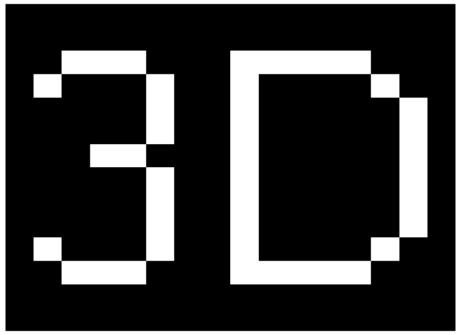

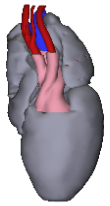

Ventral

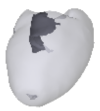

Fig. 14B

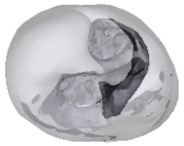

Fig. 14C

Heart Failure
Research Center
http://3d.hfrc.nl
