# Supplementary material for: Development of the Hearts of Lizards and Snakes and Perspectives to Cardiac Evolution
Source: PLoS One. 2013 Jun 5;8(6):e63651. doi: 10.1371/journal.pone.0063651 (PMC3673951; doi:10.1371/journal.pone.0063651)
Supplement: Figure S7 — 3D model of the heart of the adult ostrich ( Struthio camelus ). (PDF) [file pone.0063651.s007.pdf]

Heart of the adult ostrich

- lumen
- pulmonary vein
- left atriovetricular valve
- aortic valve
- pulmonary artery valve
- myocardium
- right ventricular free wall
- muscular flap valve
- fibro-fatty tissue
- systemic arteries
- coronaries
- pulmonary artery
- rm

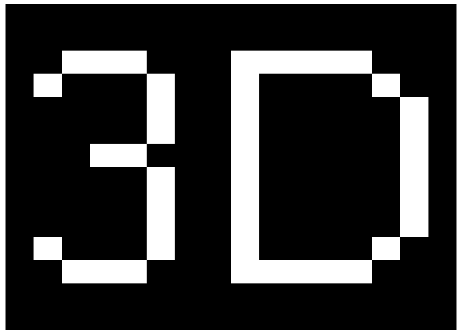

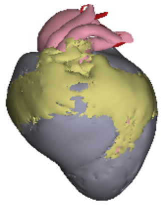

Ventral

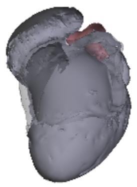

Fig. 14B

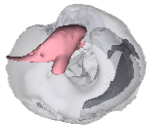

Fig. 14C

Heart Failure
Research Center
http://3d.hfrc.nl
