# Supplementary figures and images for: Development of the Hearts of Lizards and Snakes and Perspectives to Cardiac Evolution
Source: PLoS One. 2013 Jun 5;8(6):e63651. doi: 10.1371/journal.pone.0063651 (PMC3673951; doi:10.1371/journal.pone.0063651)

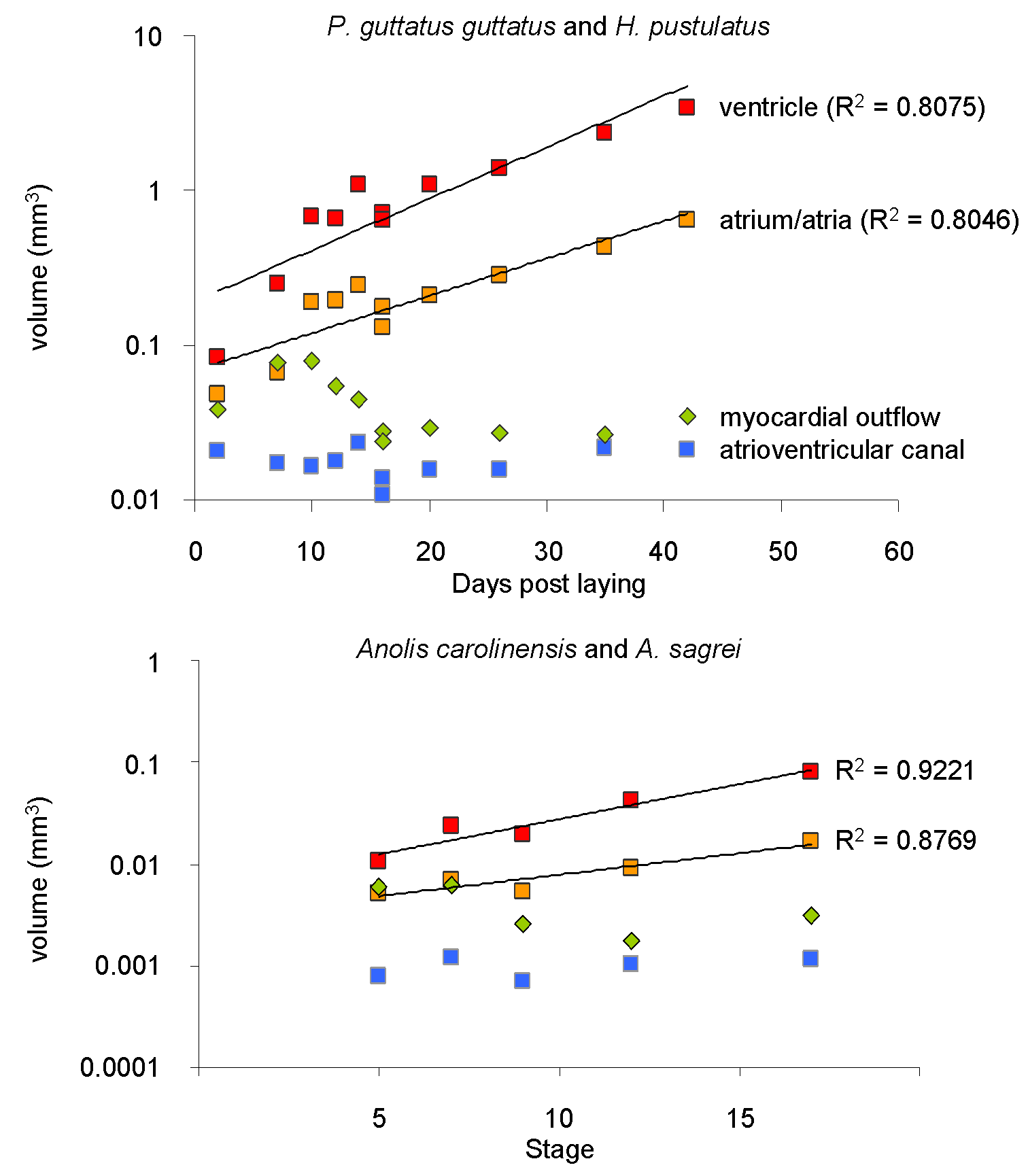

Supplement: Figure S8 — Growth of cardiac compartments. The atrial and ventricular compartments showed exponential growth, whereas there was little change in the myocardial volume of the atrioventricular canal and the myocardial outflow tract. (TIF) [file pone.0063651.s008.tif]

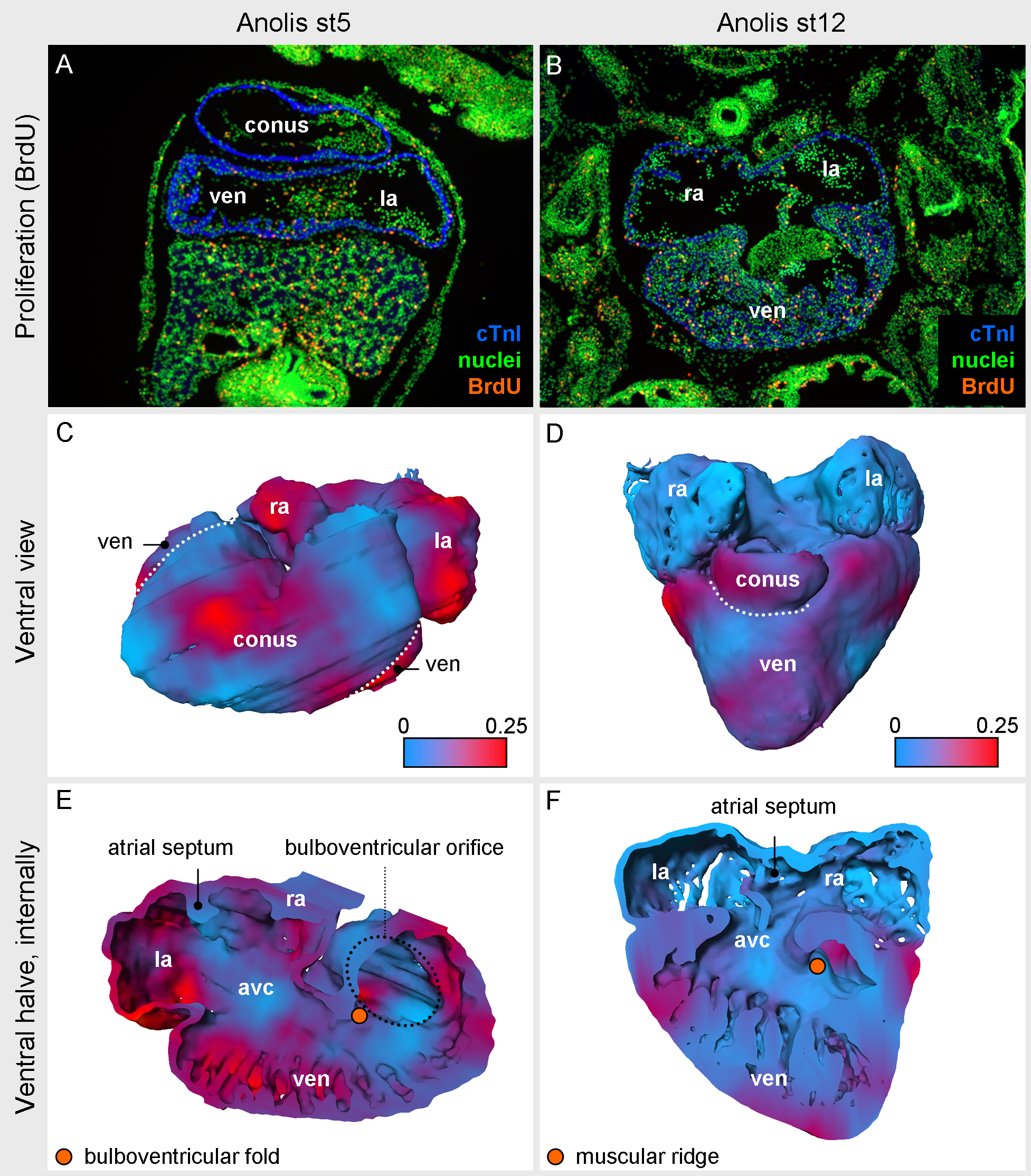

Supplement: Figure S9 — Proliferation of the hearts in two specimens of anole lizard, as assessed by BrdU incorporation. A. A 7 µm section of the st5 specimen, close to the transverse plane, showing myocardium (blue), nuclei (green) and BrdU positive nuclei (orange). B. A 7 µm section of the st12 specimen, close to the horizontal plane, showing myocardium (blue), nuclei (green) and BrdU positive nuclei (orange). C–D. Reconstructions of the myocardium upon which is projected the fraction of BrdU positive nuclei as described in [50]. The color-scale bar indicates BrdU incorporation in zero (0) to every fourth nuclei (0.25). E. Internal view of the ventral halve of the st5 specimen showing relatively high proliferation in the ballooning atria (la, left atrium; ra, right atrium) and ventricle (ven). F. Internal view of the ventral halve of the st12 specimen. At this stage the cardiac compartments almost have the proportions of the fully formed heart and proliferation is lower than in the st5 specimen, albeit the outer curvature of the ventricle still shows some proliferation. (TIF) [file pone.0063651.s009.tif]
